# Genome assembly and DNA methylation variation in an epimutant population of hybrid poplar clone NL895

**DOI:** 10.1093/plphys/kiaf415

**Published:** 2025-09-23

**Authors:** Jie He, Guang-Zheng Diao, Yang-Fan Feng, Hao-Ran Liao, Ying Guo, Li-Na Mei, Fang-Fang Fu, Tongming Yin, Fuliang Cao, Liang-Jiao Xue

**Affiliations:** State Key Laboratory of Tree Genetics and Breeding, Co-Innovation Center for Sustainable Forestry in Southern China, Key Laboratory of Tree Genetics and Biotechnology of Educational Department of China, Key Laboratory of Tree Genetics and Silvicultural Sciences of Jiangsu Province, Nanjing Forestry University, 159 Longpan Road, Nanjing 210037, China; State Key Laboratory of Tree Genetics and Breeding, Co-Innovation Center for Sustainable Forestry in Southern China, Key Laboratory of Tree Genetics and Biotechnology of Educational Department of China, Key Laboratory of Tree Genetics and Silvicultural Sciences of Jiangsu Province, Nanjing Forestry University, 159 Longpan Road, Nanjing 210037, China; State Key Laboratory of Tree Genetics and Breeding, Co-Innovation Center for Sustainable Forestry in Southern China, Key Laboratory of Tree Genetics and Biotechnology of Educational Department of China, Key Laboratory of Tree Genetics and Silvicultural Sciences of Jiangsu Province, Nanjing Forestry University, 159 Longpan Road, Nanjing 210037, China; State Key Laboratory of Tree Genetics and Breeding, Co-Innovation Center for Sustainable Forestry in Southern China, Key Laboratory of Tree Genetics and Biotechnology of Educational Department of China, Key Laboratory of Tree Genetics and Silvicultural Sciences of Jiangsu Province, Nanjing Forestry University, 159 Longpan Road, Nanjing 210037, China; State Key Laboratory of Tree Genetics and Breeding, Co-Innovation Center for Sustainable Forestry in Southern China, Key Laboratory of Tree Genetics and Biotechnology of Educational Department of China, Key Laboratory of Tree Genetics and Silvicultural Sciences of Jiangsu Province, Nanjing Forestry University, 159 Longpan Road, Nanjing 210037, China; State Key Laboratory of Tree Genetics and Breeding, Co-Innovation Center for Sustainable Forestry in Southern China, Key Laboratory of Tree Genetics and Biotechnology of Educational Department of China, Key Laboratory of Tree Genetics and Silvicultural Sciences of Jiangsu Province, Nanjing Forestry University, 159 Longpan Road, Nanjing 210037, China; State Key Laboratory of Tree Genetics and Breeding, Co-Innovation Center for Sustainable Forestry in Southern China, Key Laboratory of Tree Genetics and Biotechnology of Educational Department of China, Key Laboratory of Tree Genetics and Silvicultural Sciences of Jiangsu Province, Nanjing Forestry University, 159 Longpan Road, Nanjing 210037, China; State Key Laboratory of Tree Genetics and Breeding, Co-Innovation Center for Sustainable Forestry in Southern China, Key Laboratory of Tree Genetics and Biotechnology of Educational Department of China, Key Laboratory of Tree Genetics and Silvicultural Sciences of Jiangsu Province, Nanjing Forestry University, 159 Longpan Road, Nanjing 210037, China; State Key Laboratory of Tree Genetics and Breeding, Co-Innovation Center for Sustainable Forestry in Southern China, Key Laboratory of Tree Genetics and Biotechnology of Educational Department of China, Key Laboratory of Tree Genetics and Silvicultural Sciences of Jiangsu Province, Nanjing Forestry University, 159 Longpan Road, Nanjing 210037, China; State Key Laboratory of Tree Genetics and Breeding, Co-Innovation Center for Sustainable Forestry in Southern China, Key Laboratory of Tree Genetics and Biotechnology of Educational Department of China, Key Laboratory of Tree Genetics and Silvicultural Sciences of Jiangsu Province, Nanjing Forestry University, 159 Longpan Road, Nanjing 210037, China

## Abstract

Epimutant populations represent important genetic resources for plant breeding and selection. However, the variation and dynamics of epigenomic modifications among epimutants are still elusive. In this study, we analyzed DNA methylation patterns at both whole-genome and allelic levels in an epimutant population of a model hybrid poplar NL895 (*Populus deltoides* × *Populus euramericana* cv. “Nanlin895”). Epimutants were generated through the application of 5-Azacytidine (5-Aza) during tissue culture. A haplotype-resolved assembly of NL895 was constructed to serve as a reference for epigenomic analysis. Compared to control plants, averaged DNA methylation levels across the entire genome were reduced in epimutants. The methylation patterns of epimutants exhibited high diversity in several aspects, including the number of differentially methylated regions (DMRs), distribution of DMRs in sequence contexts, and genomic features. The observed epigenomic diversity suggests stochastic effects resulting from 5-Aza treatment. At the gene level, non-expressed genes consistently displayed higher rates of methylation across all examined epimutants. Among allele-specific expressed genes (ASEGs), fold changes between parental alleles were more pronounced in allele pairs exhibiting greater disparities in DNA methylation rates. For allele-specific methylation regions (ASMRs), the differences in methylation levels were notably elevated in ASMRs overlapped with genomic structural variations (SVs). Our results provide valuable germplasm resources characterized by phenotypic variations for poplar breeding, and the dynamics of DNA methylation in hybrid poplar epimutants highlights potential clues for application of hybrid vigor.

## Teaser

Poplar individuals in an epimutant population exhibit stochastic variations in DNA methylation and plant phenotypes.

## Introduction

Construction of mutant populations provides valuable resources for germplasm innovation in agriculture and forestry. Historically, diverse physical and chemical approaches have been applied to generate plant mutants. Ionizing radiations, including X-rays, gamma rays, and fast neutrons, can induce nucleotide deletions of various sizes at a relatively low frequency (Li et al. 2017). Chemical mutagens, such as ethyl methanesulfonate, have been successfully used to randomly create genetic mutations in plants (Greene et al. 2003; Henry et al. 2014). As the development of transgenic techniques, populations of T-DNA insertions and activation tags have been widely used for both forward and reverse genetical studies (Koncz et al. 1989; Weigel et al. 2000). In addition, the advanced genome editing techniques provide the opportunity to precisely modify genomic sequences for crop improvement (Gao 2021). The CRISPR (clustered regularly interspaced short palindromic repeats)-Cas technology, has been particularly applied to create mutant populations of rice (*Oryza sativa*), wheat (*Triticum aestivum*) and maize (*Zea mays*) (Gao 2021).

In addition to genetic variations, epigenetic variations, such as variations in cytosine methylation or chromatin modification, also contribute to phenotypic diversity in plant populations (Hirsch et al. 2012). The epimutations can be generated spontaneously or through artificial induction and inherited through multiple generations (Lieberman-Lazarovich et al. 2022). Whole-genome methylome analysis of a tomato population comprising wild individuals, landraces, and cultivars have indicated that DNA methylation variation is a determining factor of metabolic diversity in leaves (Guo et al. 2023). A recent report revealed that the effects of proximal methylation QTLs (methylQTLs) closely located to non-coding RNA genes were larger than distal methylQTLs (Liu and Zhong 2024). During the process of clonal propagation, epigenetic alleles can be generated and stably inherited in regenerated plants, which are valuable for breeding of crops through grafting or tissue culture (Lieberman-Lazarovich et al. 2022). Other approaches, such as treatments of abiotic/biotic stresses and RNAi-mediated techniques can artificially create epimutants (Dalakouras and Vlachostergios 2021).

Methylation inhibitors, such as 5-azacytidine (5-Aza) and zebularine, have the capacity to induce DNA demethylation throughout the genome (Griffin et al. 2016). 5-Aza is a cytosine nucleoside analog that can be incorporated into DNA and covalently trap the methyltransferase to deplete its activity (Christman 2002). In black poplar (*Populus nigra* “N46”), the application of 5-Aza during the regeneration process from leaf explants lead to substantially epigenetic and phenotypic changes in the epimutant population (Zhong et al. 2021). In rice, plants treated with 5-AzaD exhibited significant dwarfing phenotype (Liu et al. 2022b).

The variations in DNA methylation can influence gene expression levels and plant traits. Cytosine methylation occurs in 3 sequence contexts: CG, CHG, and CHH, which are catalyzed by various enzymes (Zhang et al. 2018). Distinct patterns of gene methylation can result in different transcriptional results. In *Arabidopsis*, the mutant *mddcc* generated by knocking out 5 known DNA methyltransferases was observed with stunted growth (He et al. 2022). At single gene level, the hypomethylation in the promoter region of *RELATED TO ABSCISIC ACID INSENSITIVE3 (ABI3)/VIVIPAROUS1 (VP1) 6* (*RAV6*) in rice, resulted in the ectopic expression of *RAV6* and larger lamina inclination and smaller grain sizes in *Epi-rav6* epimutant (Zhang et al. 2015). In comparison to model plants and crops, forest trees exhibit larger genome sizes and higher genomic heterozygosity, which pose challenges and opportunities to study the interactions between DNA methylation and gene expression at allele level.

Hybrid poplar NL895 is an elite, fast-growing cultivar known for its superior wood quality. It was selected from a hybrid population between the maternal *P. deltoides* cv. I-69 and the paternal *P.*  *×*  *euramericana* cv. I-45 (Chen et al. 2018). NL895 trees are widely propagated through cuttings for planting in southern China. At the same time, it is frequently used in transgenic experiments for trait improvement and functional studies. To explore the ranges of phenotypic and epigenetic variations in epimutants and the patterns of induced hypo and hyper methylation by the application of 5-Aza, we generated an epimutant population comprising 210 independent plants and characterized the DNA methylation dynamics in hybrid poplar epimutants. Haplotype-resolved assembly of NL895 was generated as a reference for genomic analysis. The patterns of DNA methylation remodeling induced by methylation inhibitors provide clues to understand epigenetic interactions in hybrids, which may contribute to hybrid vigor.

## Results

### Generation of epimutant plants from explants of hybrid poplar NL895

**Figure 1.**
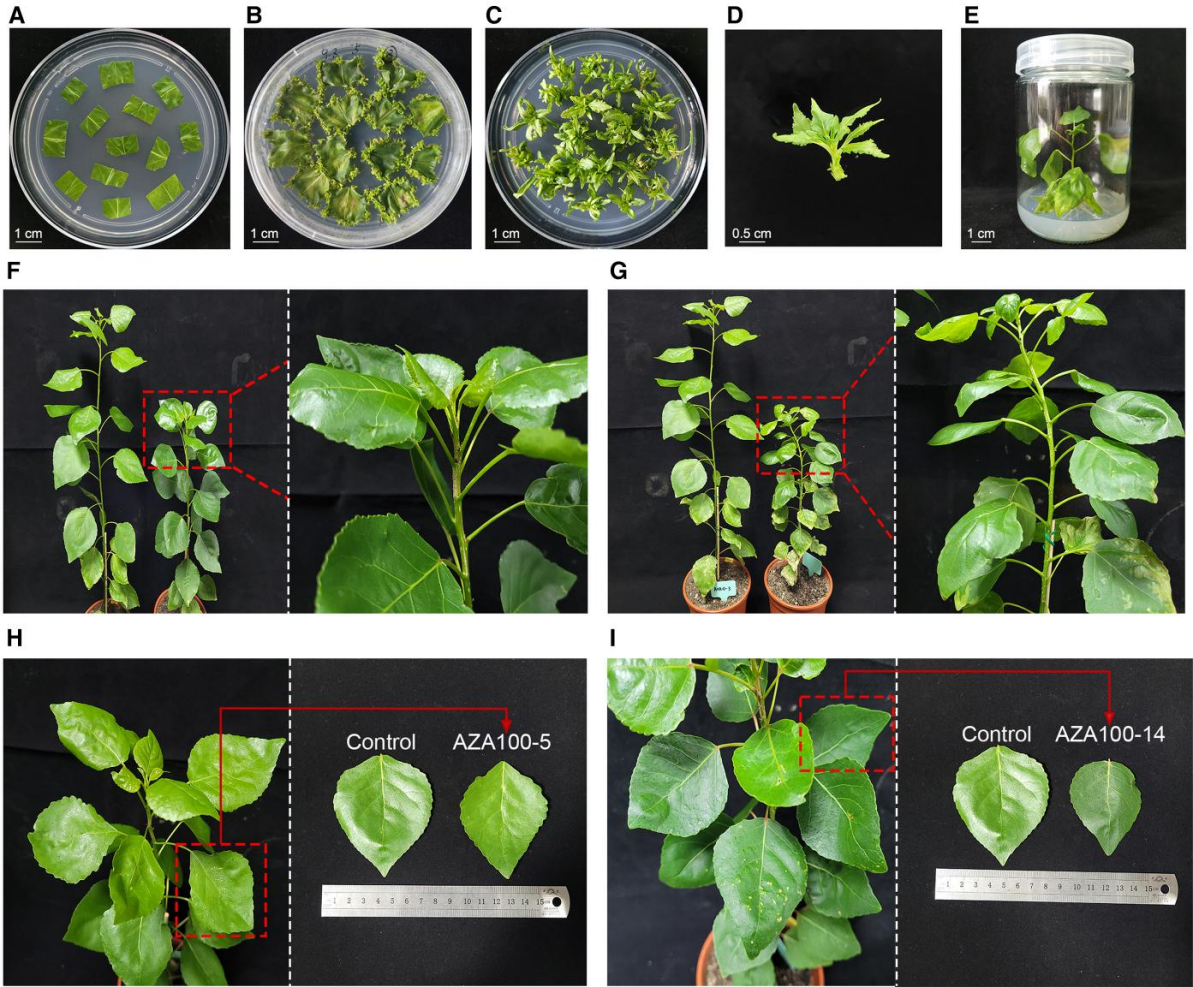
Generation of epimutant population of NL895. **A** to **E)** Process of 5-Aza treatment and stages of plant regeneration, including leaf explants **(A)**, induction of adventitious shoots **(B)**, shoot elongation **(C)**, adventitious shoot **(D)**, regenerated plants **(E)**. **F)** Clustered apical leaves in clone AZA25-7. **G)** Short internode length of clone AZA50-11. **H)** Cuneate leaf bases in clone AZA100-5. **I)** Leathery leaves in clone AZA100-14. Phenotypes of mutant leaves are highlighted with dashed boxed. The same plant and leaf are shown on the left as the control in each image.

Epigenetic mutant populations were generated through treatment of 5-Aza with different concentrations during tissue culture of NL895 leaf explants (Fig. 1, A to E). A dose-dependent effects on differentiation rates were observed for variated 5-Aza concentrations. In comparison to mock treatment with no 5-Aza, the differentiation rate increased by 2.94% to 5.23% by treatment with 5 to 25 *μ*M 5-Aza. However, shoot differentiation rates were inhibited by application of 5-Aza at concentrations ranging from 50 to 1000 *μ*M. No regenerated plants were produced on induction media containing 5-Aza at concentrations of 200 and 1000 *μ*M (Supplementary Table S1).

A total of 210 epigenetic mutants were generated, among which 15 exhibited obvious phenotypic variations, such as altered leaf shapes and colors (Supplementary Table S2). Four epimutants were selected for further molecular analysis, including AZA25-7 with clustered leaves at shoot apex (Fig. 1F), AZA50-11 exhibiting wrinkled leaves and shortened internodes (Fig. 1G), AZA100-5 and AZA100-14 with leathery leaves of reduced size (Fig. 1, H, I). Leave samples were applied for Bisulfite sequencing and transcriptome sequencing to explore DNA methylation and gene expression patterns of selected epimutants at whole-genome level.

### Assembly and evaluation of NL895 genome

Haplotype-resolved genome of hybrid poplar NL895 were assembled as a reference for genomic data analysis. A total of 547 Gb PacBio HiFi reads (89.5X) were sequenced and applied for assembly using a trio binning approach. Genomic resequencing data of 2 parental clones, I-69 and I-45, were utilized to guide the separation of HiFi reads into 2 haplotypes. The assembly sizes of maternal haplotype (hap1) and paternal haplotype (hap2) were 399.39 mb (36 contigs) and 400.52 mb (52 contigs), respectively (Table 1).

**Table 1.** Assembly and annotation statistics of the poplar NL895 genome

Statistic	Sequencing
Raw PacBio HiFi data (Gb)	35.8
PacBio HiFi sequencing depth (×)	89.5
Average reads length (bp)	19,557
Reads N50 (bp)	20,632

	Haplotype 1	Haplotype 2
Contig assembly		
Total number of contigs	36	52
Assembly size	399.39	400.52
N50 (bp)	17,854,836	14,678,315
N90 (bp)	10,185,775	7,296,527
Largest contig (bp)	26,232,212	25,720,453
LTR assembly index score	11.03	12.36
Scaffold assembly		
Total number of scaffolds	19	19
Assembly size	399.39	400.52
N50 (bp)	22,038,363	21,341,940
N90 (bp)	14,804,744	14,678,315
BUSCO completeness (%)	97.7	97.7
Annotation		
GC content (%)	33.79	33.87
Repeat content (%)	38.33	37.66
Number of protein-coding genes	38,080	37,036
Average length of protein-coding gene (bp)	3224.17	3290.12
Average length of CDS (bp)	1152.09	1179.55
Average number of exons per transcript	5.2	5.3

**Figure 2.**
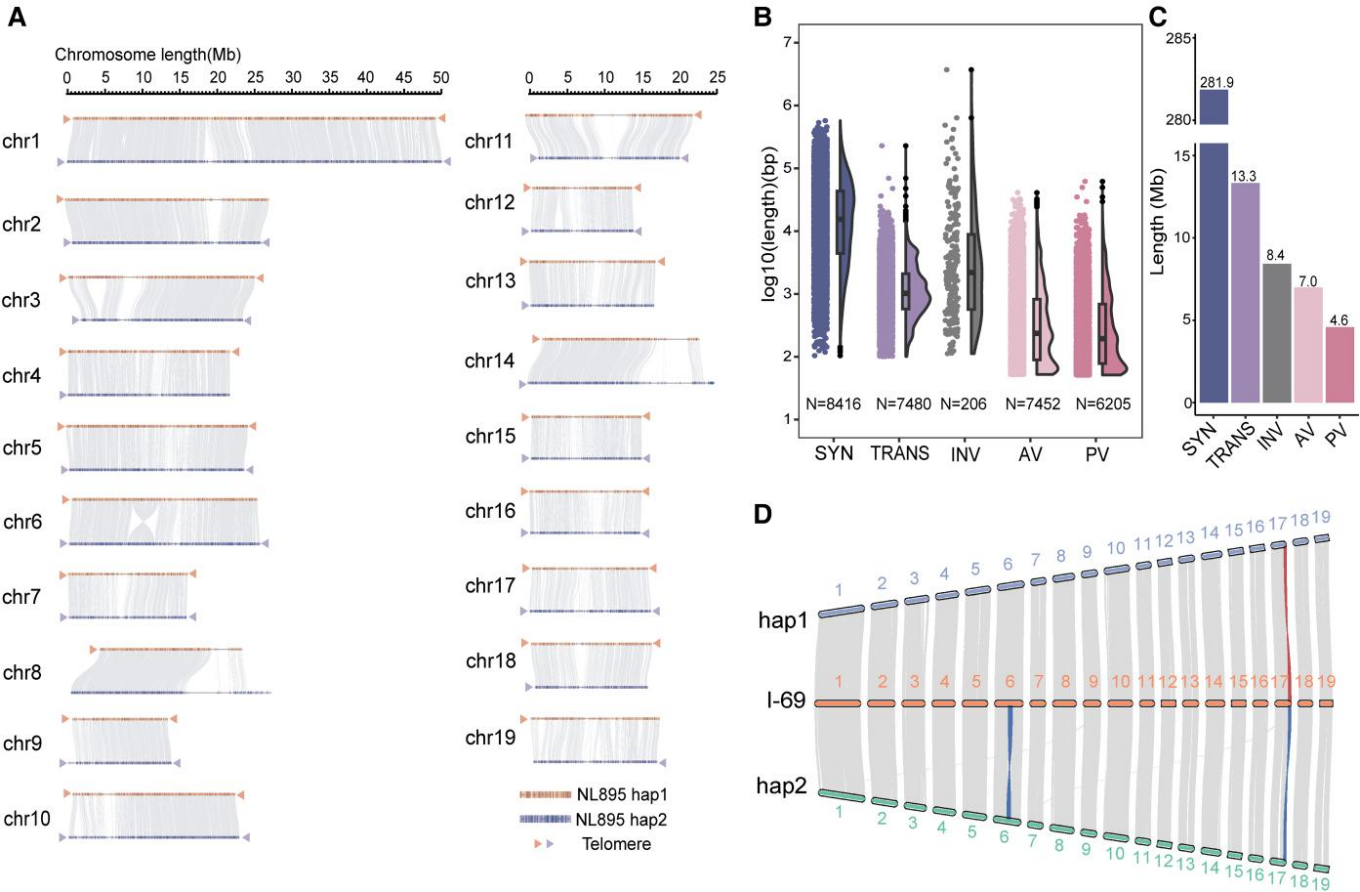
Genomic features and structural variations between 2 haplotypes. **A)** Syntenic plot of 2 haplotypes. The syntenic blocks were linked by gray lines. The colored triangles at ends of chromosomes indicated the presence of telomeric repeats. **B)** Size distributions of structural variations (SVs) between 2 haplotypes. SYN, syntenic regions; TRANS, translocated regions and inverted translocated regions; INV, inverted regions; AV, absent variations; PV, present variations. N represented the number of SVs in each category. In the boxplots, the center lines represent the medians, the box limits correspond to the 25th and 75th percentiles, the whiskers extend to 1.5 times the interquartile range, and individual points indicate outliers. The distributions of the sizes are shown in the half violin plot. **C)** Total length of SVs in hap1 assembly. **D)** Syntenic plot of 2 haplotypes versus I-69. The blue and red lines indicated the inversions of chromosome fragments in hap1 and hap2, respectively.

The assembly quality and completeness of 2 haplotypes were evaluated using BUSCO scores and long-terminal repeat (LTR) assembly index (LAI). The completeness scores from BUSCO analysis exceeded 97% for both haplotypes (Table 1). The majority of LAI scores were higher than 10 and averaged at 12, indicating a high continuity of genome assembly (Supplementary Fig. S1). In addition, 31 and 32 telomeres were detected for hap1 and hap2, respectively (Fig. 2A). Fifteen centromeric regions were predicted in hap1, and 16 ones were predicted in hap2. (Supplementary Figure S2). Majority (13) of chromosomes were assemblies at telomere-to-telomere (T2T) level for each haplotype (Fig. 2A, Supplementary Tables S3 and S4).

### Annotation of repetitive elements and protein-coding genes for NL895 genome

About 38.33% (153.09 mb) of hap1 assembly and 37.66% (150.83Mb) of hap2 assembly were annotated as repetitive sequences using combined homology and de novo approaches (Supplementary Table S5). A total of 38,080 and 37,046 protein-coding genes were further annotated for hap1 and hap2, respectively. The averaged gene lengths are 3224.17 bp and 3290.12 bp in 2 haplotypes (Table 1). About 92.02% and 94.04% of the protein-coding genes were assigned predicted functions through homolog search for haplotypes (Supplementary Table S6).

In a recent report, the genome of NL895 was also assembled and the monoploid sequences was released (Luo et al. 2024). The syntenic analysis between our assembly and the monoploid 1 indicated that both hap1 and hap2 exhibited high collinearity with the monoploid version, supported by the large portions (more than 87%) of syntenic genes with 1:1 depth pattern (Supplementary Fig. S3, A to D). Meanwhile, the BUSCO scores of both hap1 (96.1%) and hap2 (96.9%) were higher than the published monoploid assembly (94.6%) (Supplementary Table S7).

### Genomic structure variations between 2 haplotypes

As poplar NL985 is an inter-species hybrid cultivar, the sequences of 2 haplotypes exhibited significant genomic variations (Supplementary Figs. S2A and S4). In the synteny and rearrangement analysis between 2 haplotypes using SyRI, a total of 21, 343 structural variations (SVs) were detected, comprising 7480 translocation regions (TRANS, 13.3 mb), 206 inversion regions (INV, 8.4 mb), 7452 absent variations (AV, 7.0 mb) and 6205 present variations (PV, 4.6 mb) (Fig. 2, B, C). In addition, 7,727,425 SNPs were detected between 2 haplotypes (Supplementary Table S8). These SVs was validated through the coverage of circular consensus sequence (CCS) reads supporting the breakpoint of each SV. On average, each breakpoint was supported by more than 46 CCS reads, including 24 high-confidence reads (with length longer than 20 kb) (Supplementary Data Set 1), indicated the high quality of SVs detection.

Two large inversions were detected between our assembly and the genome of maternal cultivar I-69 (Fig. 2D). The inversion between hap2 and I-69 on chr6 may represent the divergence between *P. deltoides* and *P.*  *×*  *euramericana*. The inversion shared by hap1 and hap2 on chr17 may originate from the variants between 2 haplotypes of I-69. The inversions were well supported by the contigs (Supplementary Figs. S5 and S6) and raw HiFi reads (Supplementary Fig. S6, B and C). The reference-grade genome assembly and the annotated SVs provide valuable resources for functional genomics studies at multiples levels.

### Dynamic DNA methylation landscape of epimutants

**Figure 3.**
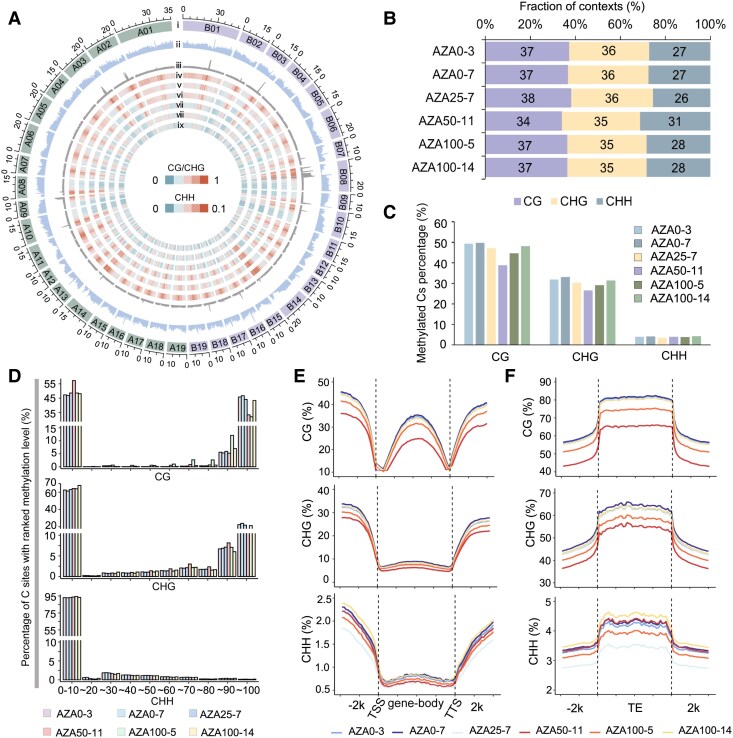
Genome-wide DNA methylation landscape of epimutants. **A)** Genome-wide profiles of DNA methylation in CG, CHG, and CHH contexts. ⅰ, chromosome length; ⅱ, gene density; ⅲ, repetitive sequences density; ⅳ and ⅴ, methylation level in CG contexts of clones AZA0-7 (ⅳ), and AZA50-11 (ⅴ); ⅵ and ⅶ, methylation level in CHG contexts of AZA0-7 (ⅵ) and AZA50-11 (ⅶ); ⅷ and ⅸ, methylation level in CHH contexts of AZA0-7 (ⅷ) and AZA50-11 (ⅸ). The statistics were performed in bins of 200-kb. **B)** Distributions of methylated sites in CG, CHG and CHH contexts in all analyzed samples. **C)** Methylation levels of Cs in CG, CHG, and CHH contexts. The values represent percentages of methylated sites in total Cs in the whole genome in 3 contexts. **D)** Distribution of C sites in 10 ranked methylation levels in CG, CHG, and CHH contexts. **E** and **F)** Regional DNA methylation levels of genes **(E)** and TEs **(F)** in CG, CHG, and CHH contexts. The fragments of gene bodies were divided into 40 bins, and upstream and downstream 2 kb regions were divided into 20 bins.

Genome-wide bisulfite sequencing (WGBS) was performed to evaluate DNA methylation levels in epimutants and control plants (Supplementary Table S9). The results indicated that AZA50-11 exhibited pronounced hypomethylation, and the other epimutants showed moderate variants in DNA methylation (Fig. 3). At the whole-genome level, higher DNA methylation was observed in genomic regions enriched with repetitive sequences, and reduced methylation levels can be found in AZA50-11, especially in gene-rich regions (Fig. 3A). Among the total methylated cytosine (mCs) sites, about 37%, 36%, and 27% were detected in control plants in CG, CHG, and CHH contexts, respectively. However, lower portion of CG (34%) and higher portion of CHH (31%) were measured in AZA50-11 (Fig. 3B). The methylation levels in 3 contexts also variated in epimutants. In CG and CHG contexts, AZA50-11 exhibited the most decrease among all the epimutants, reduced by 10.78% and 5.88% in methylation level in CG and CHG contexts, respectively (Fig. 3C). The DNA methylation levels at CG and CHG contexts exhibited polarized distributions, with approximately 40.8% and 20.4% showing high methylation level (>90%), and 49.4% and 63.8% showing low methylation level (<10%). Whereas over 90% of CHH loci exhibited low methylation levels (< 10%). Obvious increase of CG context with low methylation level was observed for AZA50-11 (Fig. 3D).

We subsequently examined the averaged methylation levels within the gene-body and flanking regions across 3 contexts. In all plants, the methylation levels were low at the transcription start sites (TSSs) and the transcription termination sites (TTSs) but higher in the upstream 2k and downstream 2k regions. The methylation levels in gene-body were high in CG context, rather than in CHG and CHH contexts (Fig. 3E). In gene-body regions, significant hypomethylation was detected in AZA50-11 in CG context, and nearly all epimutants exhibited hypomethylation in CHG and CHH contexts (Supplementary Fig. S7). The methylation level of TEs was higher than in gene-body regions, and hypomethylation patterns of epimutants on TEs were similar to protein coding genes (Fig. 3F), especially for LTR and LINE elements (Supplementary Fig. S8).

### Diverse hypomethylated and hypermethylated DMRs in epimutants

**Figure 4.**
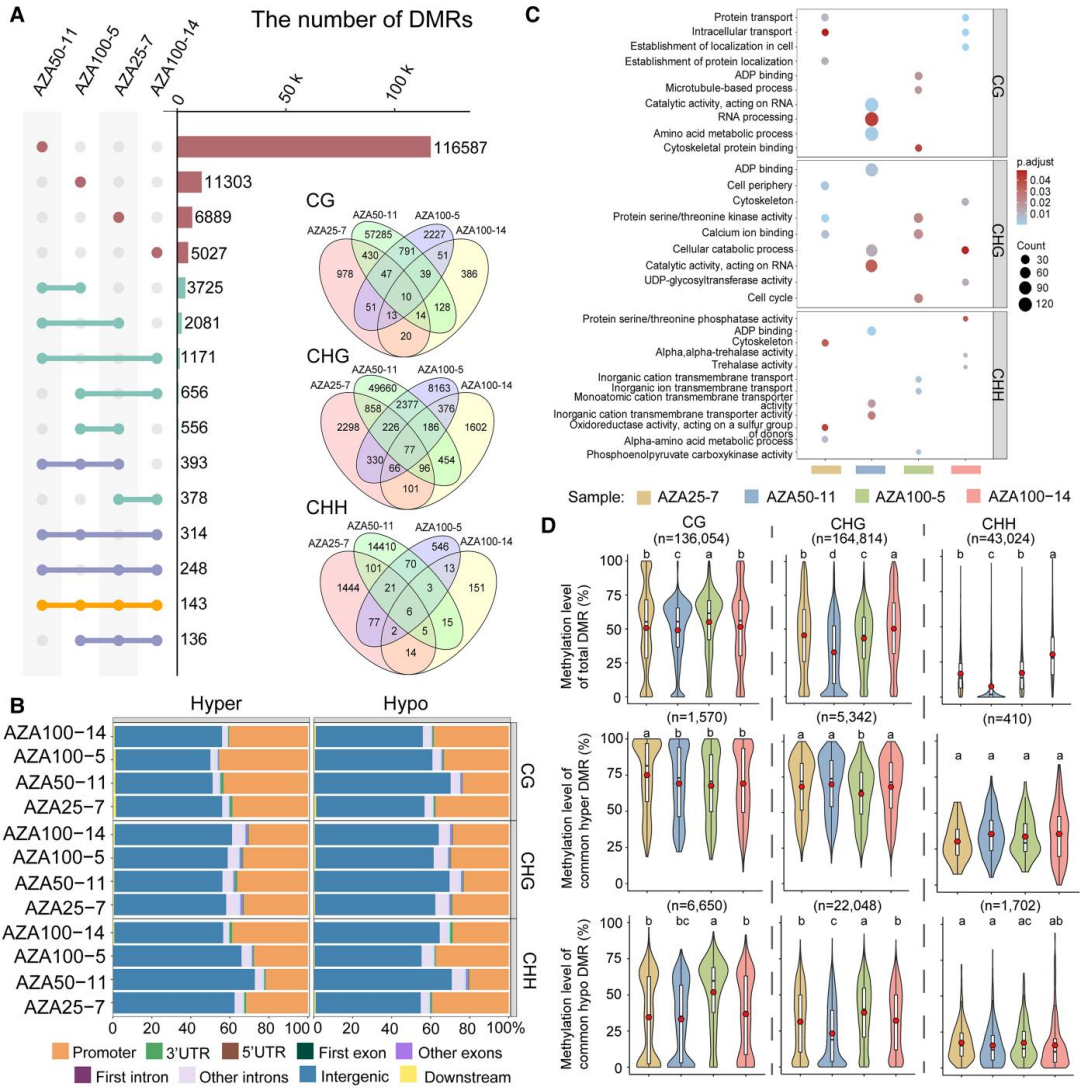
Comparative DNA methylation analysis of epimutants. Sample AZA0-7 serves as a control in the statistics. **A)** Intersection patterns of DMRs across 4 epimutants. Horizontal bars with colored nodes in the Upset plot denote shared DMR subsets, with the inset quantifying shared hypomethylated DMRs (hypo-DMRs) in CG, CHG, and CHH contexts. **B)** Proportions of hyper (left) and hypo (right) DMRs across regions of diverse genomic features. **C)** Functional enrichment analysis of genes overlapping with hypo-DMRs. **D)** Distributions of methylation levels of DMRs in epimutants in 3 contexts. Upper panel, total DMRs; Middle panel, hyper DMRs; Bottom panel, hypo DMRs. Distinct letters indicate statistically divergent groups (Mann–Whitney U test, 2-sided, *P* < 0.01). In the violin plots, red dots indicate the means, the center lines represent the medians, the box limits correspond to the 25th and 75th percentiles, the whiskers extend to 1.5 times the interquartile ranges, the shapes indicate the data distributions.

Differentially methylated regions (DMRs) between epimutants and control samples were detected in 3 contexts (CG, CHG, and CHH). The variations in epimutants were further explored in numbers of distinct and overlapped DMRs (Fig. 4A), the distributions of DMRs across diverse genomic features (Fig. 4B), enrichment of genes overlapped with DMRs (Fig. 4C), and the methylation levels of common hypomethylated DMRs (Fig. 4D).

The epimutant AZA50-11 was distinct from other plants with more than 144,000 DMRs detected, of which 93% were hypo-DMRs in comparison to AZA0-7 (Fig. 4A, Supplementary Table S10). The numbers of DMRs ranged from 10 to 21 K in the other 3 epimutants, with percentages of hypo-DMRs ranged from 50% to 89% (Supplementary Table S10). Among all the DMRs, only 143 (0.08%) DMRs were detected in all epimutants (Fig. 4A), indicating the extreme diversity of DMRs in epimutants induced by 5-Aza treatment.

The distributions of DMRs in genomic features also differed among epimutants. The DMRs of all samples were mainly distributed in intergenic and promoter regions (Fig. 4B). In CG and CHG contexts, the percentages of hyper-DMRs at intergenic regions were comparable among 4 epimutants. However, the percentage of hypo-DMRs in intergenic regions for AZA50-11 was significantly higher in CG and CHG contexts compared to other plants. In CHH context, large variations of DMRs at intergenic regions were observed for both hyper-DMRs and hypo-DMRs. The variations in numbers of DMRs in gene space agreed with the divergent patterns from GO enrichment analysis for DMGs in epimutants (Fig. 4C).

The averaged values and distributions of methylation levels at common DMRs (share by at least 2 epimutants) exhibited significant variations across detected epimutants (Fig. 4D). Among the total DMRs, the methylation levels were lowest in AZA50-11 in all 3 contexts, which is also true for common hypo-DMRs in CG and CHG contexts. The variations of methylation levels in common hyper-DMR were reduced, especially in CHH context (Fig. 4D).

The general patterns of DMRs between epimutants and AZA0-3 were similar to AZA0-7 as control. When the 2 regenerated plants without 5-Aza application were compared directly at DNA methylation level, about 30 K DMRs were also obtained (Supplementary Fig. S9, Supplementary Table S11).

### Intersections of differentially expressed gene and differentially methylated regions

**Figure 5.**
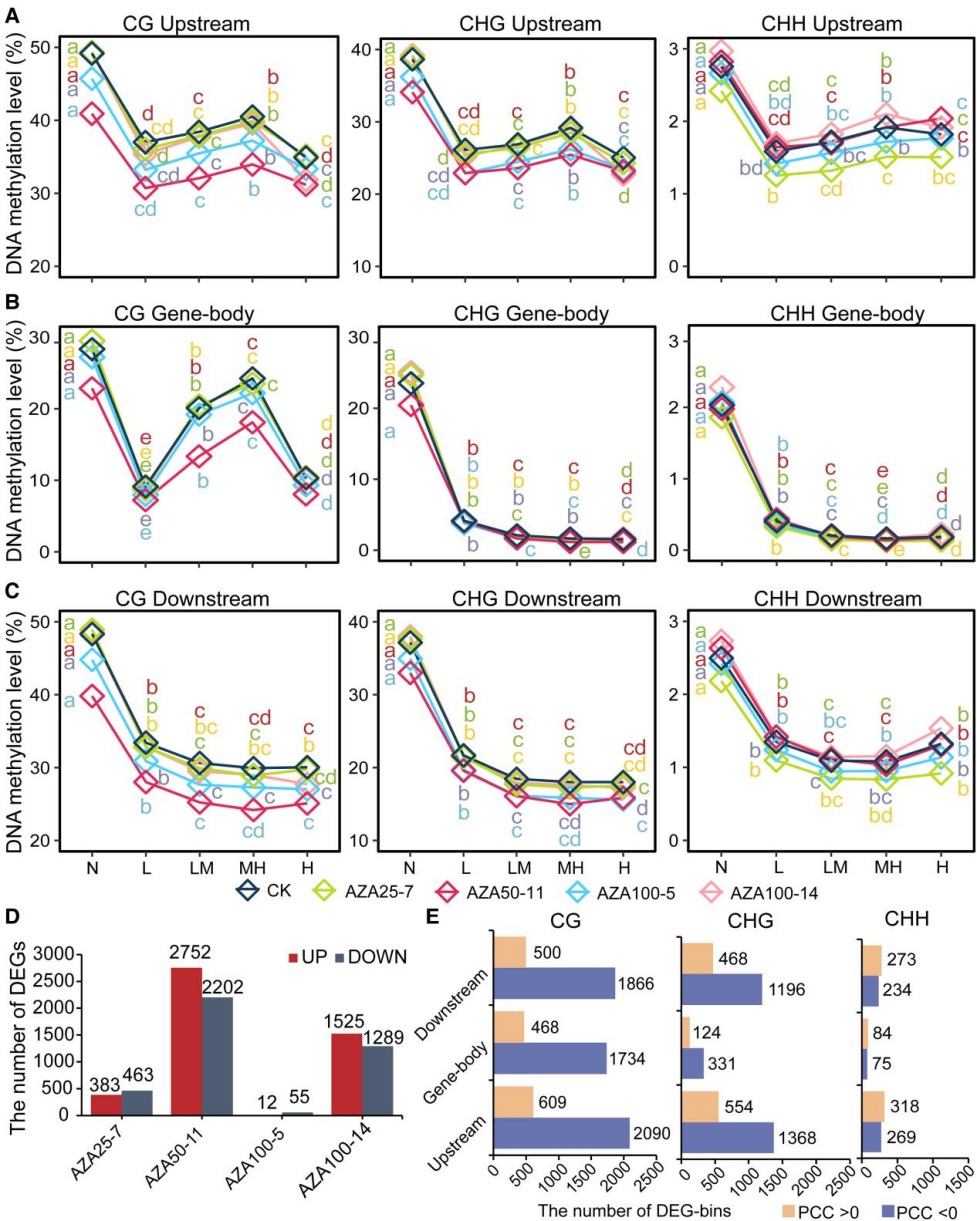
Intersections between DNA methylation and gene expression. **A** to **C)** DNA methylation levels in upstream **(A)**, gene-body **(B)**, and downstream **(C)** regions of genes with 5 ranked expression levels in epimutants and control plants (CK). Letters denote statistically distinct groups (Wilcoxon. test, *P* < 0.01). **D)** Numbers of differentially expressed genes (DEGs) in epimutants compared to the control group. **E)** Numbers of bins in gene space showing positive or negative correlations between DNA methylation levels and expression levels of the corresponding gene. The bins were defined as same as in Fig. 3E.

To characterize how the perturbation of DNA methylation affects the levels of gene expression in epimutants, we examined the relationship between them in detail. The intersections between DNA methylation and gene expression exhibited patterns dependent on methylation contexts and genomic features. Non-expressed genes (N group) were observed with highest DNA methylation level in all categories and all plants (Fig. 5, A to C, Supplementary Fig. S10A). At gene-body regions in CG context, lower methylation levels were observed for low-expressed (L) and high-expressed (H) genes, and the methylation levels were higher for low-medium-expressed (LM) and medium-high-expressed (MH) genes (Fig. 5B, Supplementary Fig. S10A). The trends were similar for upstream regions in all 3 contexts, although the extend of variation in DNA methylation were reduced (Fig. 5B). The methylation level decreased gradually as the increase of gene expression in the remaining conditions (Fig. 5, A to C, Supplementary Fig. S10A). The fractions of sites with high methylation level (HML) also variated among gene groups with ranked expression levels (Supplementary Fig. S10B).

Differentially expressed genes (DEGs) between epimutants and control plants were enriched in DMR-overlapped genes (DMGs). Among the 67 to 5000 detected DEGs in 4 epimutants (Fig. 5D), significantly enrichment in DMGs were observed in AZA50-11 for all 3 contexts. In epimutant AZA25-7, DEGs were enriched with DMGs in CG and CHH contexts. Whereas DEGs were enriched with DMGs in CHG and CHH contexts in AZA100-14 (Supplementary Table S12). Majority of the DEGs were negatively correlated with DNA methylation levels. In CG and CHG contexts, the numbers of negatively correlated bins were 2 times to 4 times higher than positively correlated bins. In CHH context, a relative limited bins were detected, and correlation trends were reverse to the other contexts (Fig. 5E).

The presence of TEs also affect the relationships between gene expression and DNA methylation. We measured the fluctuations of DNA methylation along the gene spaces using the Pearson Correlation Coefficient (PCCs) between expression levels of each gene and the methylation levels of bins along the same gene. The DNA methylation patterns were stable cross the gene space in CG context. However, the patterns exhibited large fluctuations in CHG and CHH contexts for gene containing no TEs (gene-non-TEs). For genes containing TEs (gene -TEs), the fluctuations in CHG and CHH contexts were reduced (Supplementary Fig. S10C).

### Allele-specific gene expression and methylation in epimutants

**Figure 6.**
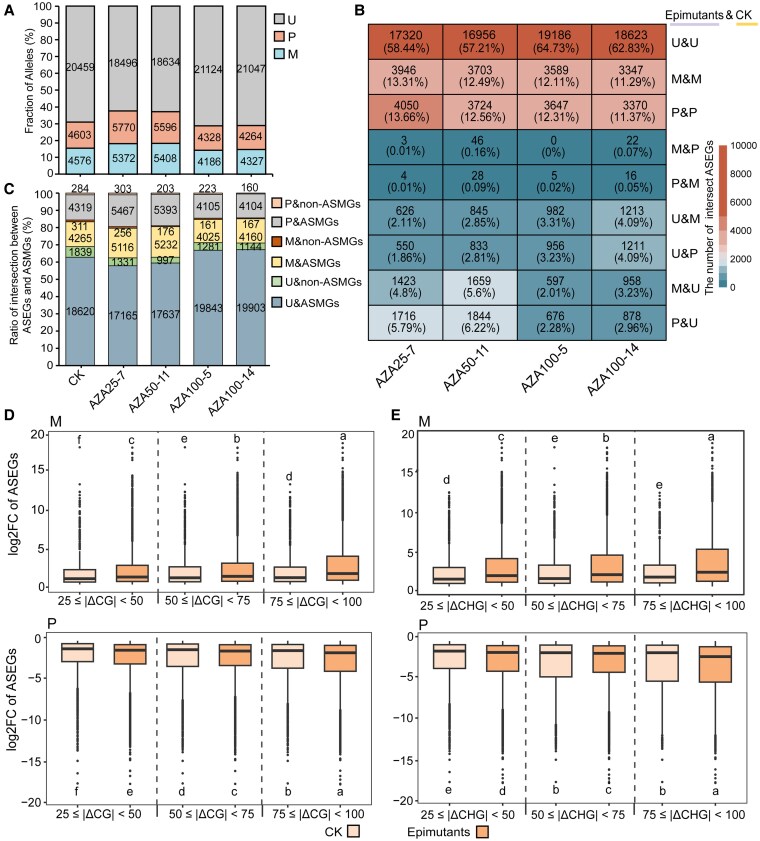
Correlation between allele-specific expression (ASE) and allele-specific methylation (ASM). **A)** Numbers of maternal-biased (M), paternal-biased (P), and unbiased (U) alleles in CK and epimutants. **B)** Comparison of biased expression patterns of alleles between epimutants and CK. The alleles were grouped into 9 categories based on the combined biased trends (M, P, or U) in epimutants (before “&”) and CK (after “&”). Heatmap represents the numbers of alleles in distinct categories. **C)** Numbers of genes in categories of combined patterns in allele-specific expression and allele-specific DNA methylation. M, P, and U indicate expression bias between alleles. ASMGs and non-ASMGs indicate genes (allele pairs) with and without biased DNA methylation, respectively. **D** and **E)** Fold changes in expression levels between paired alleles alongside distance of methylation rates on allele-specific methylated bins at 3 grades of divergence (25≤|ΔCG/CHG |<50, 50≤|ΔCG/CHG|<75, 75≤|ΔCG/CHG|<100) in CG **(D)** and CHG **(E)** contexts. Letters denote statistically distinct groups (Wilcoxon. test, *P* < 0.01). In the boxplots, the center lines represent the medians, the box limits correspond to the 25th and 75th percentiles, the whiskers extend to 1.5 times the interquartile range, and individual points indicate outliers.

Allele-biased gene expression patterns were stable between epimutants and control plants at whole-genome level. The genomic heterozygosity in inter-species hybrid poplar confers an excellent system to study gene expression and DNA methylation using allele-specific approaches. Among the 29638 allele pairs identified between 2 haplotypes (Supplementary Data Set 2), ranges of 28.7% to 37.6% alleles were detected as biased allele-specific expressed genes (ASEGs) in CK and 4 epimutants (Fig. 6A). About 60%, 12%, and 12% allele pairs were consistently un-, maternal-, and paternal-biased, respectively between epimutants and control plants. Very limited numbers of allele pairs exhibited reversed bias pattern (Fig. 6B). Majority of gene pairs showing different biased expression patterns between epimutants and controls were uniquely present in each epimutants (Supplementary Fig. S11A).

In contrast to non-ASEGs, ASEGs in all plants harbored more allele-specific DMRs (ASMR). Nearly all the allele pairs were detected to overlap with at least 1 ASMRs (Fig. 6C). The proportions of alleles with ASMR ≥ 5 and the averaged numbers of ASMRs were higher in ASEGs than in non-ASEGs (Supplementary Fig. S11, B and C). For allele pairs of ASEG, as the increase in distance of methylation rates in CG and CHG contexts (|ΔCG|, |ΔCHG|), the biased degree (log2FC) also significantly increased in epimutants and control plants (Fig. 6, D and E). In CHH context, increased biased degrees were observed between epimutants and CK for gene groups of 15≤|ΔCHH|<35 and 35≤|ΔCHH|<55 (Supplementary Fig. S12).

### Allele-specific gene expression and methylation is correlated with SVs

**Figure 7.**
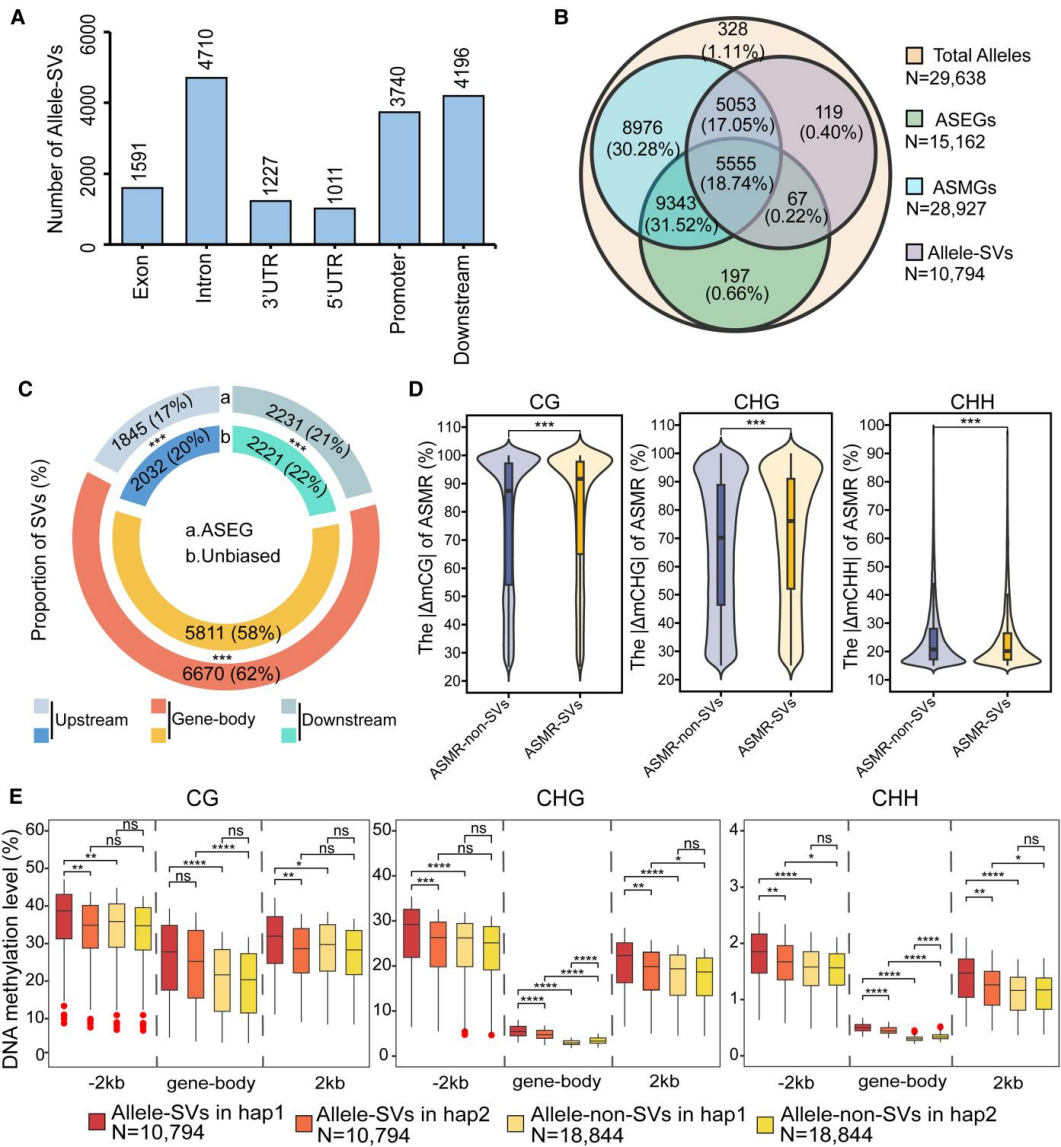
Integrative analysis of genomic structural variations (SVs) and allele-specific gene regulation. **A)** Numbers of identified SVs located at regions of paired alleles. **B)** Venn diagram showing the overlapping patterns of ASEGs, ASMGs, and Allele-SVs (alleles containing SVs). The outermost circle indicated total alleles. **C)** Proportions of SVs located at gene-body, upstream, and downstream regions of ASEGs and unbiased allele pairs. Chi-square test: ***: *P* < 0.001. **D)** Divergence of methylation levels between ASMR-SVs (ASMRs overlapping with SVs) and ASMR-non-SVs (ASMRs not overlapping with SVs) in 3 contexts. Wilcoxon rank-sum tests: ns: *P* ≥ 0.05; *: *P* < 0.05; **: *P* < 0.01; ***: *P* < 0.001; ****: *P* < 0.0001. In the violin plots, the center lines represent the medians, the box limits correspond to the 25th and 75th percentiles, and the whiskers extend to 1.5 times the interquartile range. **E)** DNA methylation levels in gene-body, upstream and downstream regions of Allele-SVs and Allele-non-SVs from 2 haplotypes in 3 contexts. The statistical test and elements of the box plots are same as in Fig. 6D.

To explore the potential effects of genomic structure variations (SVs) on allele-specific gene expression and methylation, we identified alleles overlapping with SVs between 2 haplotypes. Majority of SVs present on paired alleles located at regions of intron (5654 SVs), promoter (4977 SVs) and downstream 2 kb (5367 SVs) (Fig. 7A). Among the genes overlapping with SVs, majority were also identified as ASMGs (genes overlapped with at least 1 ASMR). Meanwhile, about 5560 and 5050 genes were detected as ASEGs and non-ASEGs (unbiased expressed genes), respectively (Fig. 7B). The proportions of SVs located at gene-body regions in ASEGs were significantly higher than in unbiased allele pairs (Fig. 7C).

We further detected the distances of methylation rate between each bin of ASMR pairs. In CG and CHG contexts, ASMR-SV (ASMRs overlapping with SVs) exhibited significantly larger distances than ASMR-non-SV (ASMRs with no overlapping SVs) (*P* < 0.001). In contrast, the distance of CHH methylation rate in ASMR-SVs were lower than ASMR-non-SVs (*P* < 0.001; Fig. 7D). The methylation levels also exhibited significant difference between alleles overlapped with and without SVs in gene-body regions (Fig. 7E). In CHG and CHH contexts, significantly different methylation levels between alleles from 2 haplotypes were also detected for SV- overlapping alleles (Fig. 7E).

## Discussion

### Genomic and germplasm resources for poplar functional genomic studies

Hybrid poplar NL895 is an elite cultivar with high adaptation to warm area in southern China (Chen et al. 2018) and a model plant extensively utilized in transgenic experiments (Zhang et al. 2013; Liu et al. 2022a). The high-quality haplotype-resolved genome assembly of NL895 provides a valuable genomic resource for functional genomic researches, such as references for analysis of multi-omics data. The genome can also be applied to design guide RNAs for gene editing and epigenetic editing of 2 haplotypes to generate novel poplar germplasms for trait improvement. As the development of peptidomics, the high-quality genome sequences will facilitate to characterize the compositions and dynamics of non-conventional peptides (NCPs) in NL895 (Guo et al. 2024).

The epimutant population of NL895 provides germplasm resources for poplar breeding and further mechanistic studies of environmental adaptations at epigenomic level. Previous reports indicated that there is no significant difference in DNA methylation between donor plants and the propagated individuals through cuttings in *P. deltoides* and *Populus trichocarpa* (Dewan et al. 2018), indicating the epigenetic stability of poplar clones. Diverse developmental phenotypes were observed for epimutants in our generated population (Fig. 1). Further screening on growth and/or physiological traits could identify germplasms for diverse breeding targets. The population size could be increased for epigenome-wide association study (EWAS) to detect epi-alleles determining pivotal traits (Zhao et al. 2024). In a recent study, multigenerational cold stress was subjected to cold-sensitive rice, resulting in the identification of a cold-tolerant line with acquired stable inheritance. The cold-tolerant trait can be inherited for at least 5 generations under normal temperature without cold treatment (Song et al. 2025). The epigenomic polymorphisms in our population endow the opportunity for multigenerational screening to apply the inheritance of acquired characteristics, especially for improvement of adaptive traits (Song et al. 2025).

### Diverse epigenomic variations among epimutants

Large epigenomic variations were observed among studied epimutants, suggesting stochastic effects of 5-Aza treatment on DNA demethylation. The strongest alteration of whole-genome DNA methylation was observed in epimutant AZA50-11 rather than plants induced by 100 *μ*M 5-Aza, which suggested that the degree of epigenomic alteration is not linearly correlated with 5-Aza concentration. Our results also indicated that the application of low concentration 5-Aza (≤ 25 *μ*M) increases the induction rate of adventitious shoots, whereas the induction rate was inhibited by higher concentration of 5-Aza (≥ 50 *μ*M). At DMR level, the total numbers, proportions in 3 contexts, and the distributions across genomic features exhibited significant diversity in the studied epimutants (Fig. 4), indicating the stochastic effects of 5-Aza on diverse genomic contexts. The numbers of DEGs between epimutants and control plants also variate by epimutant (Fig. 5D).

The effects of 5-Aza treatment could be determined by many factors such as (epi)genetic backgrounds and ages of donor plants, plant hormones in tissue cultures, and environmental conditions. In *Arabidopsis*, explants treated with 5-Aza exhibited reduced embryogenic response (Grzybkowska et al. 2018), meanwhile the rooting processes can be promoted by 5-Aza for adult tissues rather than juvenile tissues (Massoumi et al. 2017). The application of 5-Aza altered the methylation and expression levels of diverse genes in cells of cultured tissues through interaction with (epi)genetic and environmental factors. A set of these cells succeed to go through the regeneration process. The status of DNA methylation of regenerated plant individuals could be determined by the status of the original cells.

Considering the comprehensive effects of 5-Aza treatment, diverse approaches could be applied to improve the generation efficiency of epimutants. The concentrations of 5-Aza could be optimized for poplar tissues of different (epi)genetic backgrounds. Different combinations of plant hormones and/or other epigenetic inhibitors could be applied together with 5-Aza to regulate the regeneration processes. Recent reports indicated that Trichostatin A (TSA), a histone deacetylase inhibitor, promotes the embryonic transition of the explants or root development in *A. thaliana* (Choi et al. 2023) and barley (Nowak et al. 2024). The functions of genes controlling processes of epigenomic modification and plant regeneration could be manipulated through delivering of synthetic mRNA, dsRNA, and siRNA (Yong et al. 2025), which could be applied together with 5-Aza during tissue culture to induce the generation efficiency of epimutants.

### Allele-specific expression contributed by DNA methylation and SVs

The heterosis of hybrid poplar is commonly observed, which shows that F1 hybrids are superior to the parents in growth, biomass, and stress resistance. Previous report indicated that the heterosis results from phenotypic stability of multiple dominant traits, particularly at suboptimal conditions (Zanewich et al. 2018). The dominant traits in hybrid poplar could be caused by non-additive allele specific expression in metabolism such as carbon and nitrogen pathways (Zhang et al. 2025). In epimutants and control plants of NL895, about 28% to 38% allele pairs between 2 haplotypes exhibit ASEs (Fig. 6A). In comparison to control plants, about 60% allele pairs stay unbiased expression patterns, about 24% allele pairs exhibited consistent biased patterns, and about 14% allele pairs were biased expressed in epimutants or controls only (Fig. 6A). The stability of ASEs for majority allele pairs, along with the dynamic expression of alleles in small proportions, may contribute to phenotypic stability and non-additive ASEs observed in hybrid poplar.

The stability of biased ASEs could be caused by significant (epi)genomic variations between 2 haplotypes. Among the ASEGs, an increase in expression fold-changes of 2 alleles is observed alongside a corresponding increase in the distance of methylation rates within CG and CHG contexts (Fig. 6D). In addition, higher proportion of SVs located at gene-body regions were observed in ASEGs than in unbiased genes (Fig. 7C). The distances of DNA methylation levels in CG and CHG contexts between paired alleles with SVs are also significantly higher than paired alleles without SVs (Fig. 7D). Genomic SVs between alleles could contribute to ASEs through 2 mechanisms. First, the sequence variations may disrupt or create cis-regulatory elements controlling gene expression levels. Second, the genomic variations may result in allele-specific DNA methylation and chromatin accessibility (Gheldof et al. 2013).

It has been observed that substantial DNA methylation remodeling occurs in F1 hybrids in relative to their parents (Shen et al. 2012; Zhang et al. 2016). The epigenetic interactions between parental alleles mediated by small interfering RNAs (siRNAs) are proposed to play roles in DNA methylation remodeling in hybrids (Greaves et al. 2016; Kakoulidou and Johannes 2024). The SVs present in parental alleles may impair the interactions through siRNAs. The measurement of DNA methylation levels of candidate parent clones could help to guide the combination of them to maximize hybrid performance (Dalakouras and Vlachostergios 2021). In addition, 5-Aza could be applied to treat F1 hybrids to introduce dynamics of DNA methylation, which could result in more non-additive regulation at DNA methylation and transcriptional levels, especially for clonally propagated plants such as poplar trees.

In summary, we reported the epimutant population of hybrid poplar NL895 induced by 5-Aza treatment, along with a haplotype-resolved genome assembly. The DNA methylation patterns exhibited stochastic dynamics among the epimutants. Notable phenotypic variations were observed within the epimutant population, highlighting its potential value for plant breeding applications. Monitoring methylation patterns across multiple generations through cuttings under various abiotic and biotic stresses could yield deeper insights into the interplay between epigenomic modifications and stress adaptation. The integration of additional epigenetic mutagens alongside 5-Aza may further enhance diversity in DNA methylation of hybrids, which may provide clues to understand DNA methylation remodeling for vigor of hybrid plants.

## Materials and methods

### Plant materials and mediums for tissue culture

The plants of hybrid poplar NL895 propagated from a single tree were applied in our experiments. The mediums used in the tissue culture processes include: shoot induction medium (SIM), 0.5 mg/L 6-Benzylaminopurine (6-BA), 0.002 mg/L thidiazuron (TDZ), 0.8% agar, Murashige and Skoog (MS) salts, and 3% sucrose; shoot elongation medium (SEM), 0.1 mg/L 6-BA, 0.001 mg/L TDZ, 0.8% agar, MS salts, and 3% sucrose; and root induction medium (RIM), 0.1 mg/L NAA, 0.8% agar, half-strength MS salts (1/2 mS), and 2.5% sucrose.

### Generation of epimutant population

The epimutant plants were generated through application of 5-Aza during the processes of tissue culture. The 3rd-5th leaves of 40-day-old seedlings were excised into pieces of 1 × 2 cm² and used as explants. The leaf explants (LEs) were cultured on shoot induction medium (SIM) supplemented with varying concentrations of 5-Aza (0, 5, 10, 25, 50, 100, 200, and 1000 *μ*M). A total of 12 LEs were treated with 5-Aza at each concentration, and applied with 3 replicates. The tissues were maintained for 40 days in the tissue culture room with a temperature of 25 ± 2 °C under long-day conditions (16 h light/8 h dark), and an illumination intensity of 55 *μ*mol·m⁻²·s⁻¹. The differentiation rates and the morphologies of LEs were assessed at the 35th day after shoot induction. After the SIM stage, the clustered shoots were transferred to shoot elongation medium (SEM). Subsequently, shoots measuring between 1 and 2 cm in length were transferred to root induction medium (RIM).

### Acclimatization of epimutant plants

The seedlings derived from tissue culture were then transplanted into pots filled with a mixture of soil, perlite, and vermiculite in a ratio of 2:1:1 (v/v/v). After growing in soil for 5 mo, growth traits including plant height, basal diameter, leaf length, and leaf width were measured for 10 healthy plants from each group of 5-Aza treatment. Four epimutants exhibiting dramatical phenotypic variations along with 2 control plants were selected for further analysis.

### DNA extraction and library construction for genome sequencing

The fresh leaves of a 5-mo-old NL895 individual were collected for PacBio HiFi sequencing. High-quality genomic DNA was extracted using the CTAB method (Anderson et al. 2018). Genomic DNA fragments of approximately 20 kb were prepared using g-TUBE (Covaris, USA) for library construction and subsequently sequenced using PacBio HiFi technique.

### Genome assembly of hybrid poplar NL895

Trio-binning method was applied to assemble 2 haplotypes of hybrid poplar NL895. The resequencing data of parental cultivars I-69 (maternal, *P. deltoides*) and I-45 (paternal, *P.*  *×*  *euramericana*, PRJNA430966) (Zhu et al. 2018) were utilized to guide the separating of PacBio HiFi reads. The assembly process involved 3 steps: (ⅰ) The *k*-mer frequencies of NGS reads from 2 parents were calculated using yak with parameters “count -k31 -b37”. (ⅱ) Haplotype binning of HiFi reads from NL895 guided by parental *k*-mer counts and subsequent haplotype assembly were performed using hifiasm v0.16.1 (Cheng et al. 2021). (ⅲ) Redundant contigs were removed using Purge_dups v1.2.5 (Guan et al. 2020). Non-redundant contigs were anchored onto chromosomes employing the reference genome of I-69 (Bai et al. 2021) using RaGOO v1.1 (Alonge et al. 2019).

### Evaluation of 2 haplotype assemblies

The completeness of 2 haplotype assemblies were evaluated using BUSCO v3.0.2 with parameters “-l embryophyta_odb10” (Manni et al. 2021). The continuity of assemblies were assessed using LAI values and contig N50 length, and the LAI values were calculated by LTR_retriever v1.1 (Ou and Jiang 2018).

Telomeric sequences of 2 haplotypes were identified using a Perl script searching for “TTTAGGG” motif at both ends of each chromosome. Centromeres of each chromosome were predicted using quarTeT v1.1.8 (Lin et al. 2023). The boundaries of the centrosomic regions were estimated from the frequency of tandem repeats.

### Annotation of repetitive sequences

The de novo and homology-based strategies were employed to annotate repetitive sequences for 2 haplotypes. A de novo library of repetitive sequences was constructed using RepeatModeler v1.0.11 (Flynn et al. 2020) and MITE-Hunter (Han and Wessler 2010). LTR_Finder v1.0.2 (Xu and Wang 2007) and LTRharvest v1.6.2 (Ellinghaus et al. 2008) were used to detect intact retrotransposons. In addition, LTR_retriever was applied for integrating and filtering processes to obtain a high-quality LTR library (Ou and Jiang 2018). The achieved libraries of repetitive sequences were combined with public databases including Repbase (Bao et al. 2015), Dfam (Hubley et al. 2016), and MIPS (Mewes et al. 2000) to construct an integrated transposable element (TE) library. Finally, RepeatMasker v4.0.7 (Smit 1996) was used to identify and mask genomic TE sequences based on our custom TE library. The hard-masked genome sequences were used for subsequent gene prediction.

### Annotation of protein-coding genes

An integrated strategy combining ab initio, homology-based, and transcriptome-based prediction was used to predict protein-coding genes. Augustus (Stanke et al. 2004) and GeneMark (Lomsadze et al. 2014) were utilized to train a coding gene model for ab initio predictions. Protein sequences of 5 species, namely *A. thaliana*, *P. trichocarpa*, *Oryza sativa*, *Salix Purpurea*, and *P. euphratica*, were mapped onto genome assemblies using GeMoMa v1.9.0 (Keilwagen et al. 2016) for homology-based prediction. In the process of transcriptome-based prediction, HISAT2 v.2.1.0 (Kim et al. 2015) was used to map trimmed RNA-seq reads onto the reference genome. Trinity v2.6.5 (Haas et al. 2013) and TransDecoder v5.5.0 (https://github.com/TransDecoder/TransDecoder) with default paraments were used to assemble and annotate transcripts, respectively.

EvidenceModeler (EVM) v1.1.1 and MAKER pipeline v2.31.10 were used to combine all gene prediction results into 2 consensus gene sets (Haas et al. 2008; Holt and Yandell 2011). The Gffcompare v0.11.2 (Pertea and Perțea 2020) was utilized to integrate EVM and MAKER annotations into an unified gene set, which was subsequently optimized using PASA v2.4.1 (Haas et al. 2003). Genes shorter than 50 bp or with incomplete coding regions (lacking start and stop codons) were excluded from further analysis. The sensitivity of gene predictions was estimated using BUSCO. DIAMOND v2.0.14 (Buchfink et al. 2015) was used to map protein sequences onto various databases, including NR, SwissProt, eggNOG5, Pfam, TAIR10, GO, and KEGG for functional annotation.

### Detection and validation of SVs between 2 haplotypes

The hap1 and hap2 assemblies were aligned using MUMmer v4.0.0rc1 (Marçais et al. 2018) with parameters “–mum -c 100 -l 50”, and filtered using delta-filter with parameters “-m -i 90 -l 100”. The filtered delta file was applied to detect SVs using SyRI v1.5.1 (Goel et al. 2019) with default settings. As defined by SyRI, the variations were categorized into the following types: INV (inverted region), TRANS (translocated region), INVTR (inverted translocated region), DUP (duplicated region), INVDP (inverted duplicated region), CPL (copy loss in query), CPG (cpy gain in query), INS (the query had an extra insertion sequence relative to the reference), DEL (the query had a deletion sequence relative to the reference), and SNP. For INS and DEL, the variations with length longer than 50 bp were defined as SVs, while the variations with length less than or equal to 50 bp were considered as short variations. For simplicity, the SVs were grouped into 4 major categories, INV, TRANS, AVs, and present variations (PVs) (Qin et al. 2021). Both original TRANS and INVTR were referred as TRANS; the CPL, DEL, and DUP/INVDP loss were referred as AVs; the CPG, INS, and DUP/INVDP gain were referred as PVs.

The detected SVs were validated through checking the coverage of HiFi reads at the breakpoints. CCS reads with length higher than 20 kb were considered as high-confidence reads. The alignment results were manually inspected using the integrative genomics viewer (IGV) software (Thorvaldsdóttir et al. 2013) to validate the largest inversion on chromosome 6.

### Processes of whole-genome bisulfite sequencing

Total genomic DNA was extracted from leaves of 4 epimutants (AZA25-7, AZA50-11, AZA100-5, and AZA100-14) and 2 controls (AZA0-3, AZA0-7) using the CTAB method, with 2 biological replicates for each sample. DNA samples were fragmented into sizes of ∼300 base pairs, followed by end repairing and adaptor ligating. The EZ DNA Methylation Gold Kit (D5006) was subsequently used to convert unmethylated cytosines (C) into uracils (U), followed by library construction. The insert sizes and concentrations of libraries were assessed using Agilent 2100 Bioanalyzer and StepOnePlus Real-Time PCR system. Sequencing was performed on Illumina Hiseq X10 platform.

### Data analysis of WGBS reads

The WGBS reads were processed using FastQC for quality control. The adapters and low-quality reads were removed by Trimmomatic v0.39 (Bolger et al. 2014). High-quality reads were aligned to NL895 reference genome using Bismark v0.22.3 (Krueger and Andrews 2011) for methylation calling. Unmethylated Lambda DNA was used as a control to calculate the conversion rates during bisulfite treatment. PCR duplicates were eliminated using deduplicate_bismark, and the methylation statuses of cytosines were called through bismark_methylation_extractor.

C sites with read depth ≥ 5 and FDR < 0.05 in binomial test were considered as reliable methylated sites. The methylation levels in CG, CHG, and CHH contexts were calculated as the percentage of reads supporting mCs relative to total number of reads covering the specific sites. Bins of genes and TEs were partitioned using CGmapTools v0.1.2 (Guo et al. 2018), and the methylation levels of bins were calculated using weighted methylation algorithm (Schultz et al. 2012) as below:


$$\overset{n}{\underset{i=1}{\sum }}\;C_{i}/\overset{n}{\underset{i=1}{\sum }}\;(C_{i}+T_{i})$$

*C_i_* denoted the counts of reads supporting methylated cytosines at each site (*i*), *C_i_* + *T_i_* represented the total number of covering reads at each site. The variable *i* referred to the order of each cytosine in the genomic bin, and *n* represented the total number of cytosine sites in the genomic window.

### Identification of DMRs and DMR-associated genes

The DMRs of 4 epimutants compared to CKs were identified separately using the Methylkit package v1.24.0 (Akalin et al. 2012), employing a sliding window size of 200 bp and a step size of 100 bp. Genomic windows that contain more than 5 cytosines in each context were selected for the calculation of methylation levels using the weighted calculation method described above. DMRs were defined as windows exhibiting methylation differences higher than 25%, 25%, and 15% in CG, CHG, and CHH contexts, respectively (Q-value < 0.05). Locations of DMRs on genomic features were annotated utilizing the ChIPseeker package (Yu et al. 2015). Genes overlapping with at least 1 DMR within their gene body or flanking regions were classified as differentially methylated genes (DMGs). Functional enrichment analysis of hypo-DMGs were conducted using the clusterProfiler package (Wu et al. 2021).

### Processes of RNA sequencing

Plant samples utilized for transcriptome sequencing were identical as in WGBS. Total RNA was extracted from leaves using the RNAprep pure Plant Kit (DP441, Tiangen Biotech, Beijing, China) in accordance with the manufacturer's instruction. The quality and concentration of RNA were determined using a NanoPhotometer (Implen, CA, USA) and RNA 6000 Nano Kit (Agilent Technologies, CA, USA), respectively. RNA sequencing libraries were constructed using the TruSeq RNA Library Preparation Kit v2. The obtained libraries were sequenced on Illumina HiSeq 2500 platform and paired-end reads of 150 bp were achieved.

### Differential expression analysis of epimutants and controls

Quality control of RNAseq reads were performed as for WGBS reads. High-quality clean reads were aligned onto the NL895 reference genome using STAR v2.7.9 (Dobin et al. 2013), followed by calculation of read counts of genes using featureCounts v2.0.2 (Liao et al. 2014). Gene expression abundance was measured in terms of TPM (transcripts per million) values using an R script. The DEGs were identified using DEseq2 (Love et al. 2014), applying a threshold of |log_2_ (fold change)| > 1 and FDR < 0.05.

### Intersections of methylation and expression levels of genes

The methylation levels of genes were calculated for gene sets with ranked expression levels. The genes were classified into 5 groups based on their TPM values, including N (TPM = 0), L (0 < TPM ≤ 1), LM (1 < TPM ≤ 7), MH (7 < TPM ≤ 100), and H (TPM > 100). Significance tests of DNA methylation levels among 5 groups were assessed using Wilcoxon Rank Sum Test (*P* < 0.05).

The proportions of genes with ranked methylation levels were also calculated for genes with different expression ranks. The categories of DNA methylation levels included zero methylation level (equal to 0), LML (Low Methylation Level, not higher than 10%), MML (Middle Methylation Level, higher than 10% and not higher than 20%), and HML (High Methylation Level, higher than 20%).

The enrichment of differentially methylated genes (DMGs) in sets of DEGs were tested using 1-tailed Fisher's exact test. And the correlations of gene expression levels in TPM and DNA methylation levels in percentage were calculated using Pearson correlation.

### Syntenic analysis of assemblies and characterization of allele-specific regulation

The syntenic analysis was performed using JCVI (MCScan) v1.1.19 (Tang et al. 2008). The 2 haplotypes of NL895 were compared with I-69 and published monoploid assemblies to analyze the variations between assemblies. The hap1 and hap2 assemblies were compared to identify parental allele pairs, followed by the detection of orthologous genes using the bidirectional-best-hits method and OrthoFinder (v2.5.4) software (Emms and Kelly 2019).

Read counts of RNAseq data were extracted for parental alleles, which were subsequently applied for allele-specific expression analysis using edgeR package (Robinson et al. 2010). The bins corresponding to each allele pair were analyzed with the calculateDiffMeth function in methylKit to identify ASMRs. Allele pairs overlapped with at least 1 ASMR were classified as ASMGs.

## Supplementary Material

kiaf415_Supplementary_Data

## Data Availability

The whole-genome sequence HiFi reads have been deposited in the Sequence Read Archive (SRA) database of NCBI under accession number SRR29505324. The final chromosome genome assemblies and annotation of 2 haplotypes were deposited in the CNCB database with PRJCA043198. The raw resequencing reads, including RNA sequencing data and bisulfite sequencing data have been deposited in the SRA of NCBI under accession number PRJNA1127491.

## Supplementary data

The following materials are available in the online version of this article.

**Supplementary Figure S1.** LAI scores in 19 chromosomes of 2 haplotype genomes.

**Supplementary Figure S2.** Prediction of centromere regions based on distribution with highest frequency repeat sequences in 2 haplotypes.

**Supplementary Figure S3.** Comparative analysis of 2 haplotypes and published monoploid assembly (Luo et al. 2024).

**Supplementary Figure S4.** Structural variations (SVs) between 2 haplotypes of NL895.

**Supplementary Figure S5.** Distributions of contigs on chromosomes of hap1.

**Supplementary Figure S6.** Distributions of contigs on chromosomes of hap2 and evidence of chromosome inversion on chromosome 6.

**Supplementary Figure S7.** The methylation levels of epimutants and control in gene bodies and flanking regions.

**Supplementary Figure S8.** DNA methylation levels in exon and intron of protein coding genes and 4 types of transposable elements (TEs).

**Supplementary Figure S9.** Comparative methylome analysis of epimutants revealed epigenetic divergence.

**Supplementary Figure S10.** Correlations of gene expression levels and DNA methylation levels.

**Supplementary Figure S11.** Allele-specific biased gene expression and DNA methylation in epimutants.

**Supplementary Figure S12.** Fold changes between alleles of gene locus with allele-specific methylation at 3 grades of divergence in CHH contexts.

**Supplementary Table S1.** Statistics of differentiation time and differentiation rate of leaf explants from NL895 on medium supplemented with 5-Aza.

**Supplementary Table S2.** Categories of epimutants with diverse phenotypes.

**Supplementary Table S3.** Sites and lengths of the telomeres on hap1.

**Supplementary Table S4.** Sites and lengths of the telomeres on hap2.

**Supplementary Table S5.** Repetitive elements identified in the hap1 and hap2 assemblies.

**Supplementary Table S6.** Statistics of protein-coding gene with annotated functions using diverse databases.

**Supplementary Table S7.** BUSCO analysis for annotated transcriptomes of 2 haplotypes and publishes monoploid assemblies.

**Supplementary Table S8.** Summary of identified genomic variations.

**Supplementary Table S9.** Summary of total and mapped WGBS reads.

**Supplementary Table S10.** Hyper and hypo DMRs in comparison to AZA0-7 in 3 contexts.

**Supplementary Table S11.** Hyper and hypo DMRs in comparison to AZA0-3 in 3 contexts.

**Supplementary Table S12.** The enrichment analysis of DMGs in total DEGs between epimutants and control plants.

**Supplementary Data Set 1.** Read coverage Statistics at Structural Variation (SV) breakpoints.

**Supplementary Data Set 2.** Identification of alleles between 2 subgenomes.
