# Supplementary material for: Genome assembly and DNA methylation variation in an epimutant population of hybrid poplar clone NL895
Source: Plant Physiol. 2025 Sep 23;199(2):kiaf415. doi: 10.1093/plphys/kiaf415 (PMC12501979; doi:10.1093/plphys/kiaf415)
Supplement: kiaf415_Supplementary_Data [file kiaf415_supplementary_data.zip › Supplementary Data.pdf]

Genome assembly and DNA methylation variation in an epimutant population of hybrid poplar clone NL895

He *et al.*

Supplementary Figures

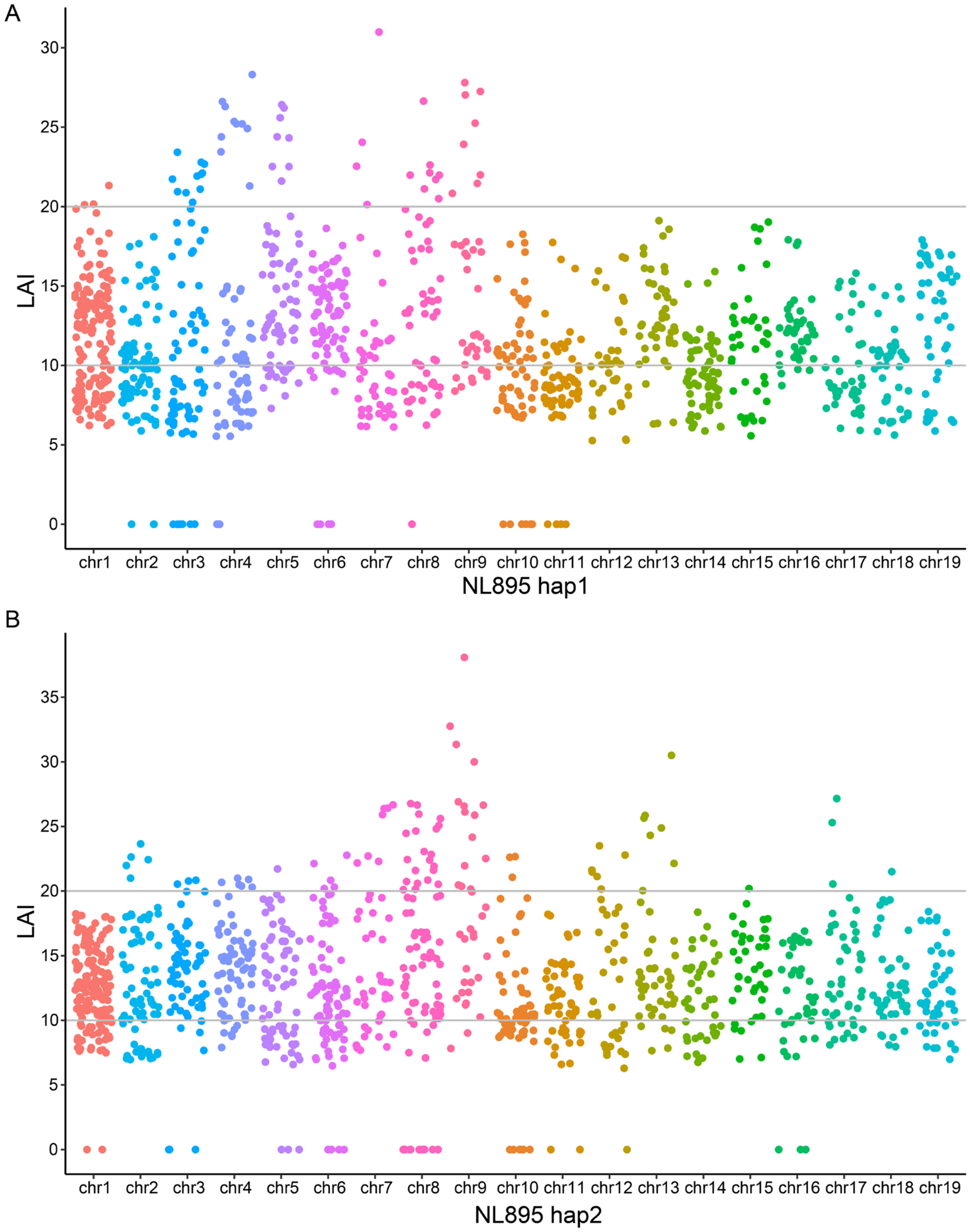

Supplementary Figure S1. LAI scores in 19 chromosomes of two haplotype genomes. The LAI scores were calculated in bins of 3 Mb in each chromosome of hap1 (A) and hap2 (B). The y-axis showed the LAI scores. The two grey lines in the plots indicated the thresholds of 10 and 20, respectively.

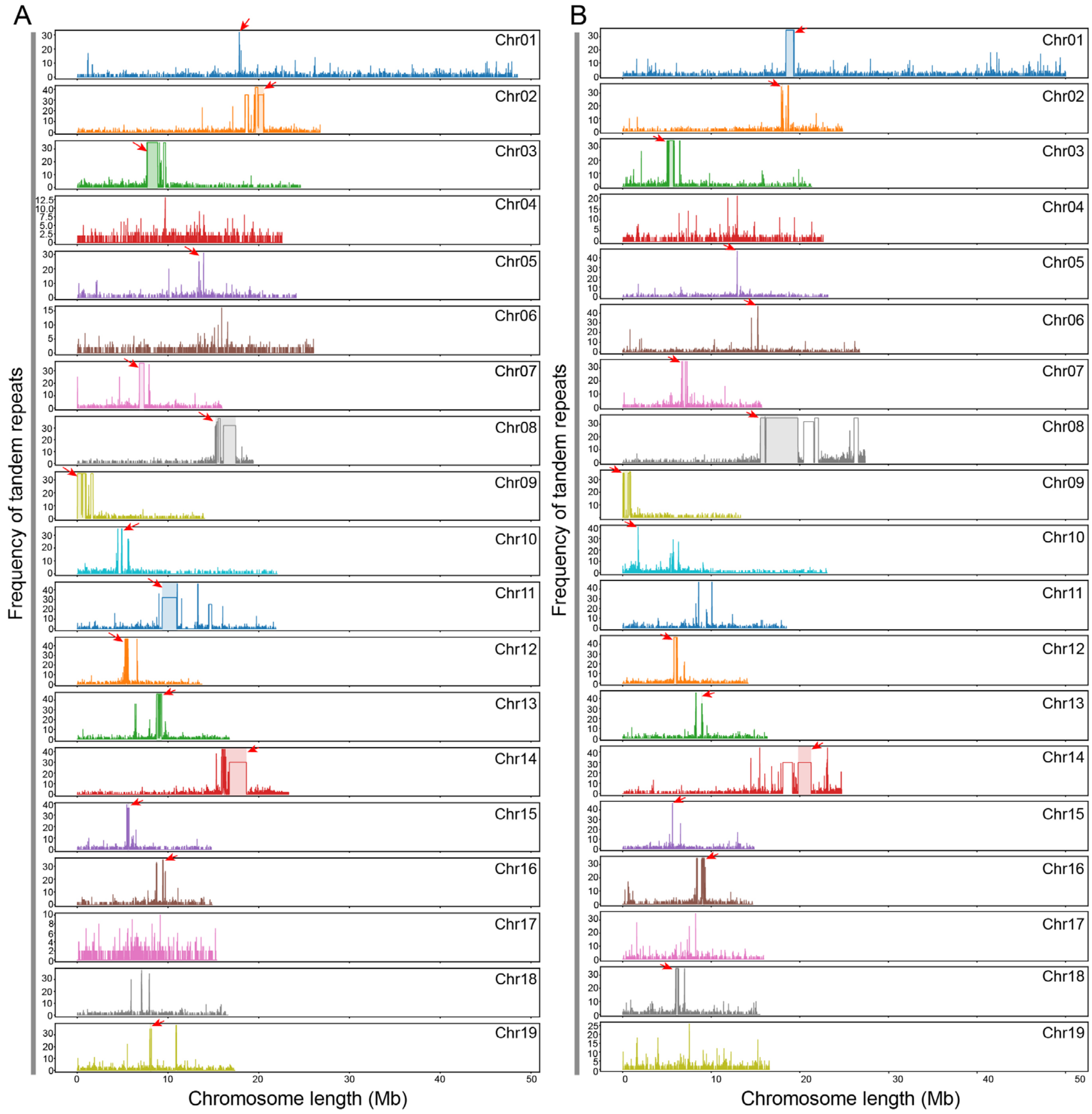

Supplementary Figure S2. Prediction of centromeric regions based on distribution with highest frequency repeat sequences in two haplotypes. (A), hap1, **(B)**, hap2. The y-axis on left indicated the frequency of tandem repeats in 1kb bins. The semi-transparent boxes and red arrows represented the potential centromeres with highest frequency repeats. The x-axis indicated the length of each chromosome.

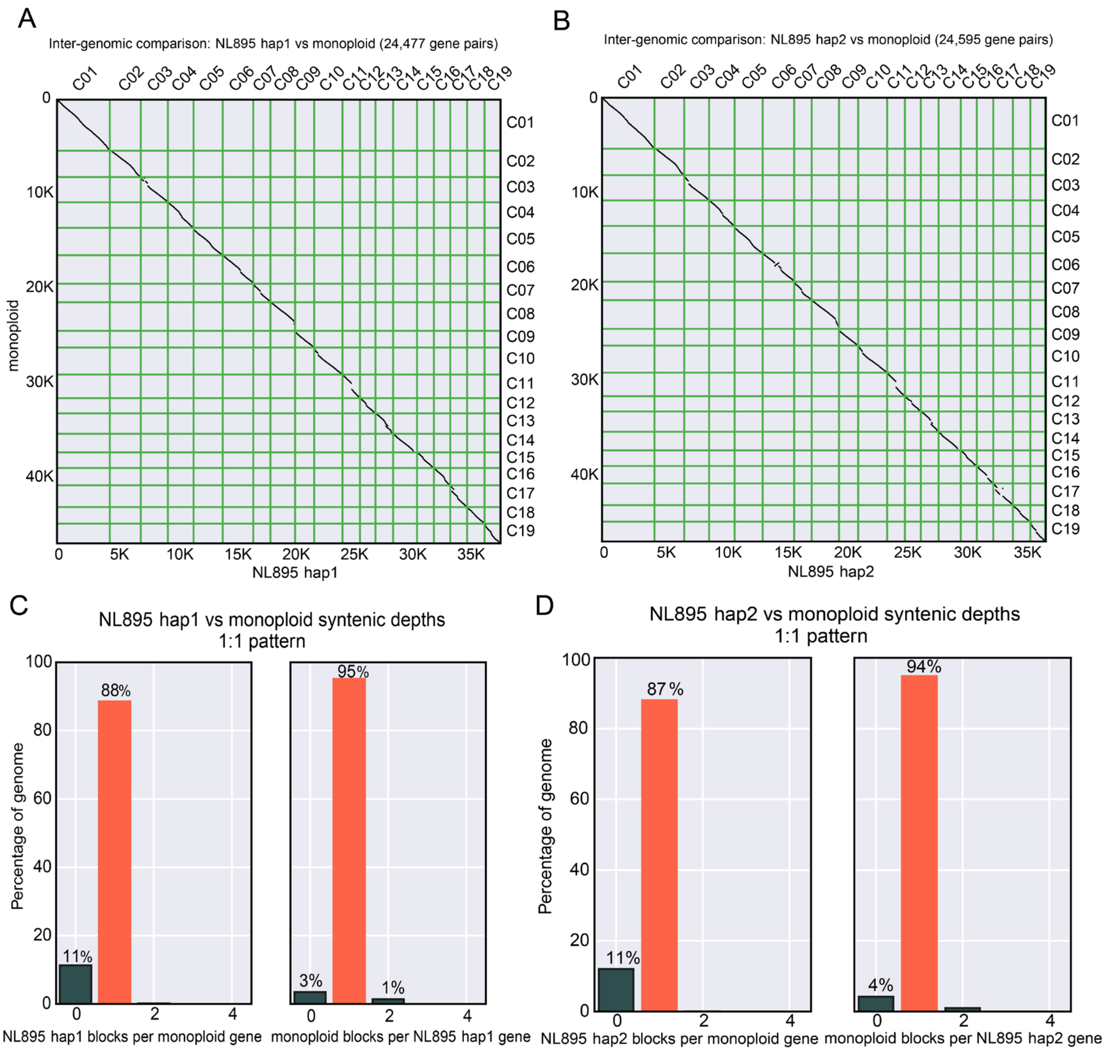

Supplementary Figure S3. Comparative analysis of two haplotypes and published monoploid assembly (Luo et al., 2024). (A-B), Synteny plots of two haplotypes versus monoploid assembly using protein sequences. **(C-D)**, The syntenic depths of two haplotypes and monoploid. The red bars represent the proportions of syntenic pairs with 1:1 pattern.

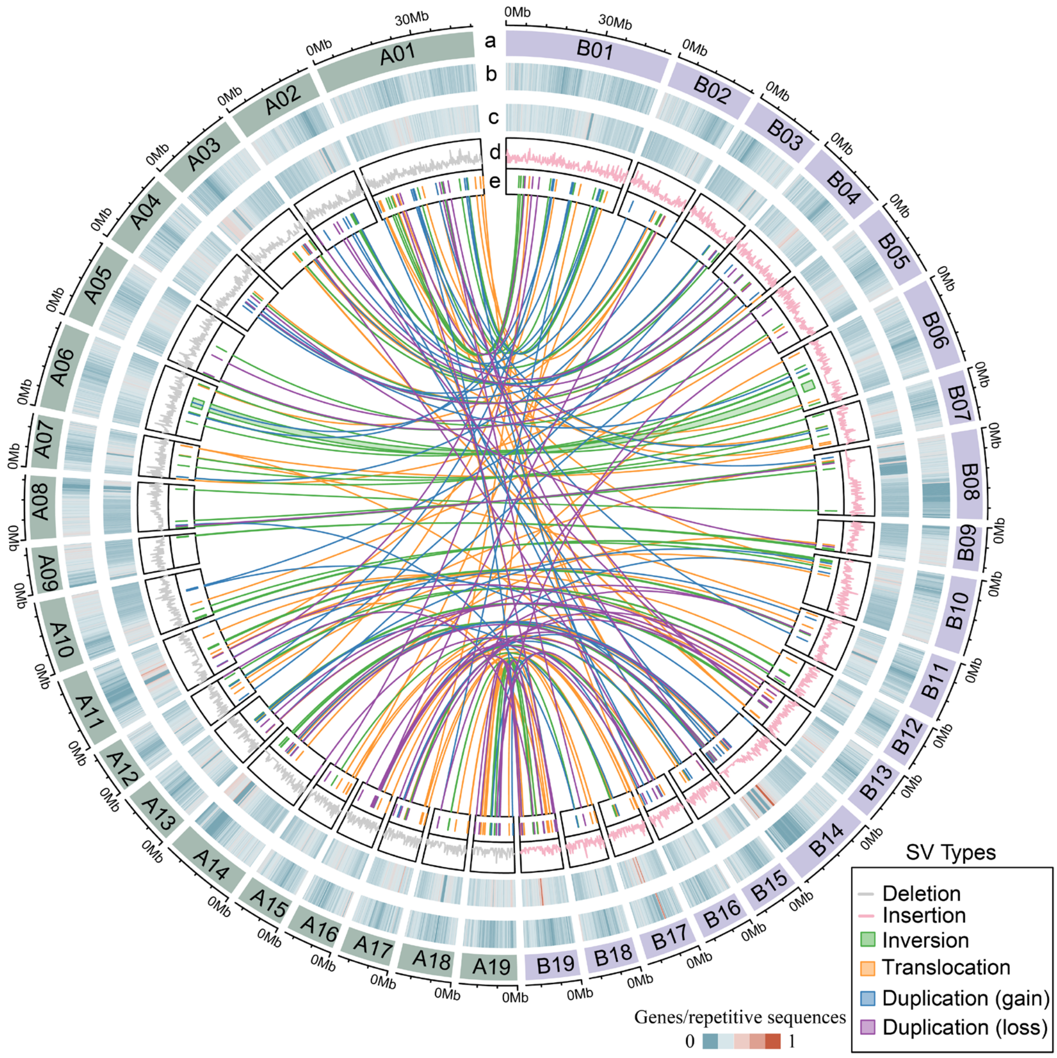

Supplementary Figure S4. Structural variations (SVs) between two haplotypes of NL895. (a) Chromosome length, (b) Density of genes, (c) Density of repetitive sequences, (d) Density of Indels (including Insertions and Deletions), (e) Distribution of SVs across the genome. The displayed SVs are larger than 5 kb.

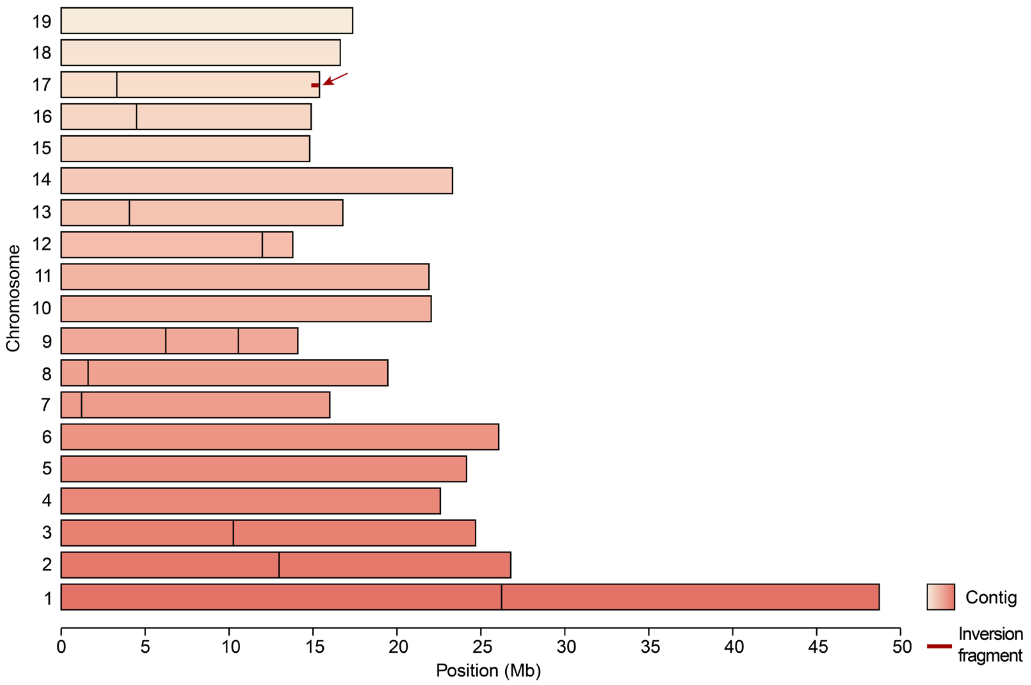

Supplementary Figure S5. Distributions of contigs on chromosomes of hap1.

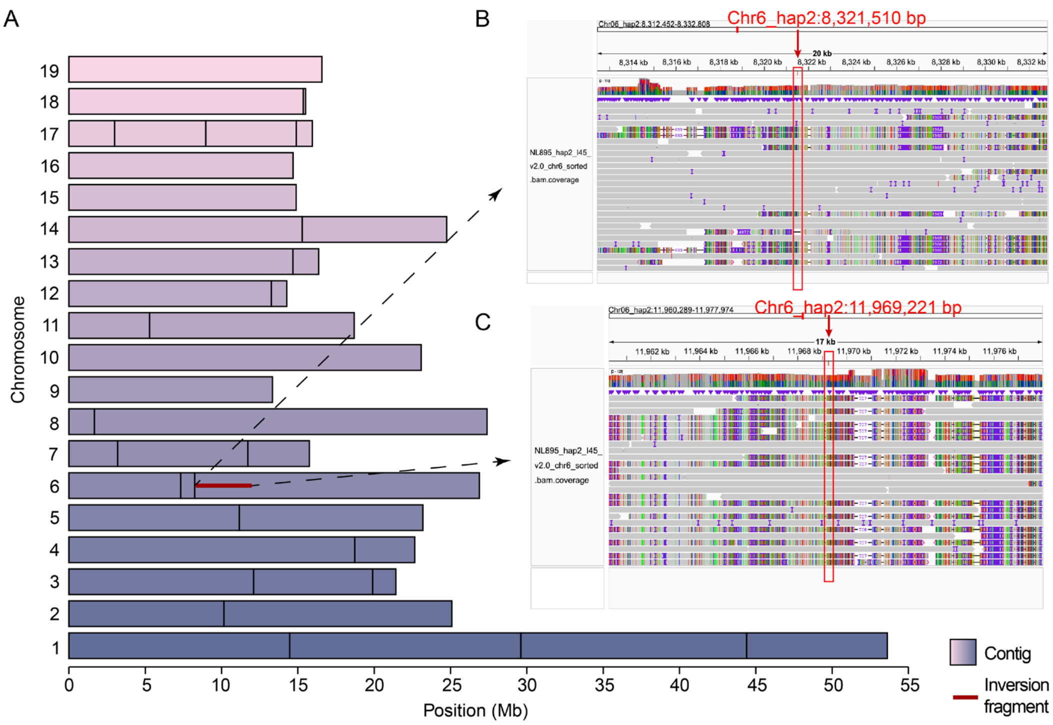

Supplementary Figure S6. Distributions of contigs on chromosomes of hap2 and evidence of chromosome inversion on chromosome 6. (**A**), Contigs on each chromosome of hap2. PacBio HiFi reads spanning over the breakpoints (arrows) of right (B) and left (C) ends of the inversion.

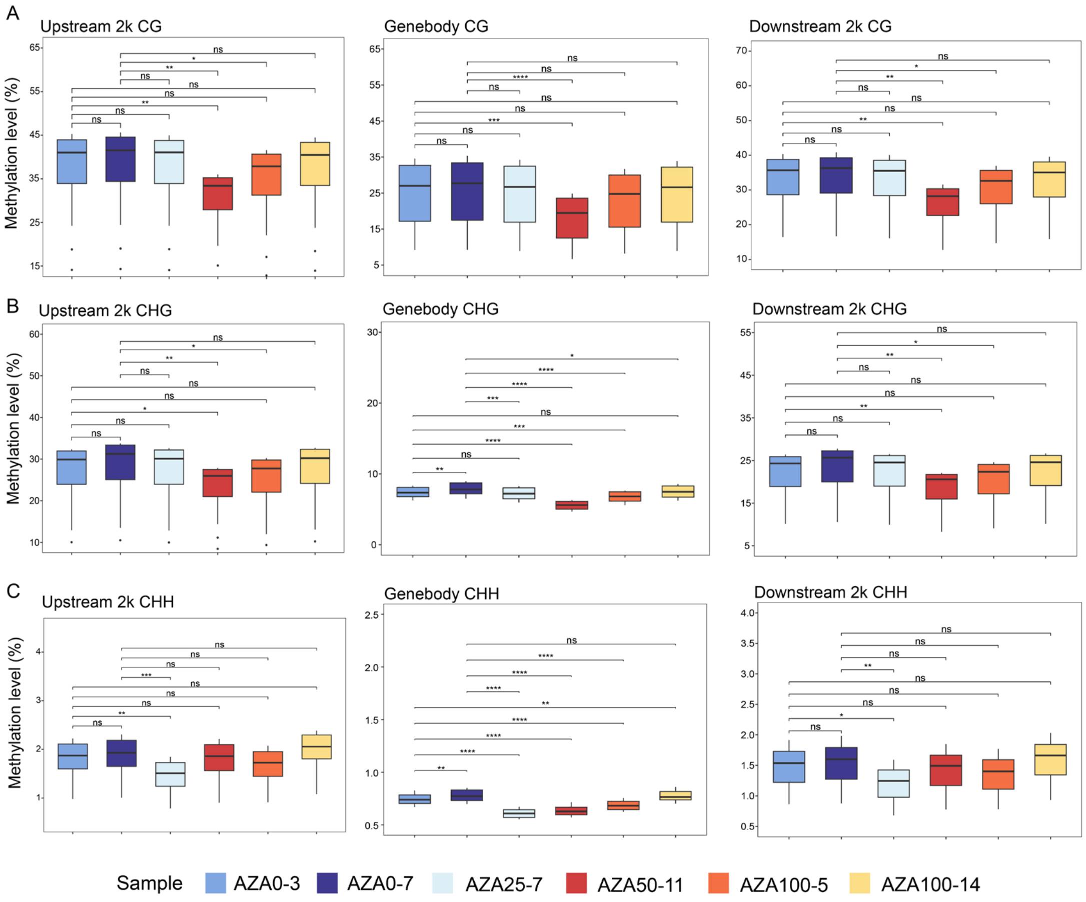

Supplementary Figure S7. The methylation levels of epimutants and control in gene bodies and flanking regions. The data was present in CG (A), CHG (B), and CHH(C) contexts, respectively. In the boxplots, the center lines represent the medians, the box limits correspond to the 25th and 75th percentiles, the whiskers extend to 1.5 times the interquartile ranges, and individual points indicate outliers. p values were calculated by Wilcoxon rank sum test, ns: $p > 0.05$; *: $p < 0.05$; **: $p < 0.01$; ***: $p < 0.001$; ****: $p < 0.0001$.

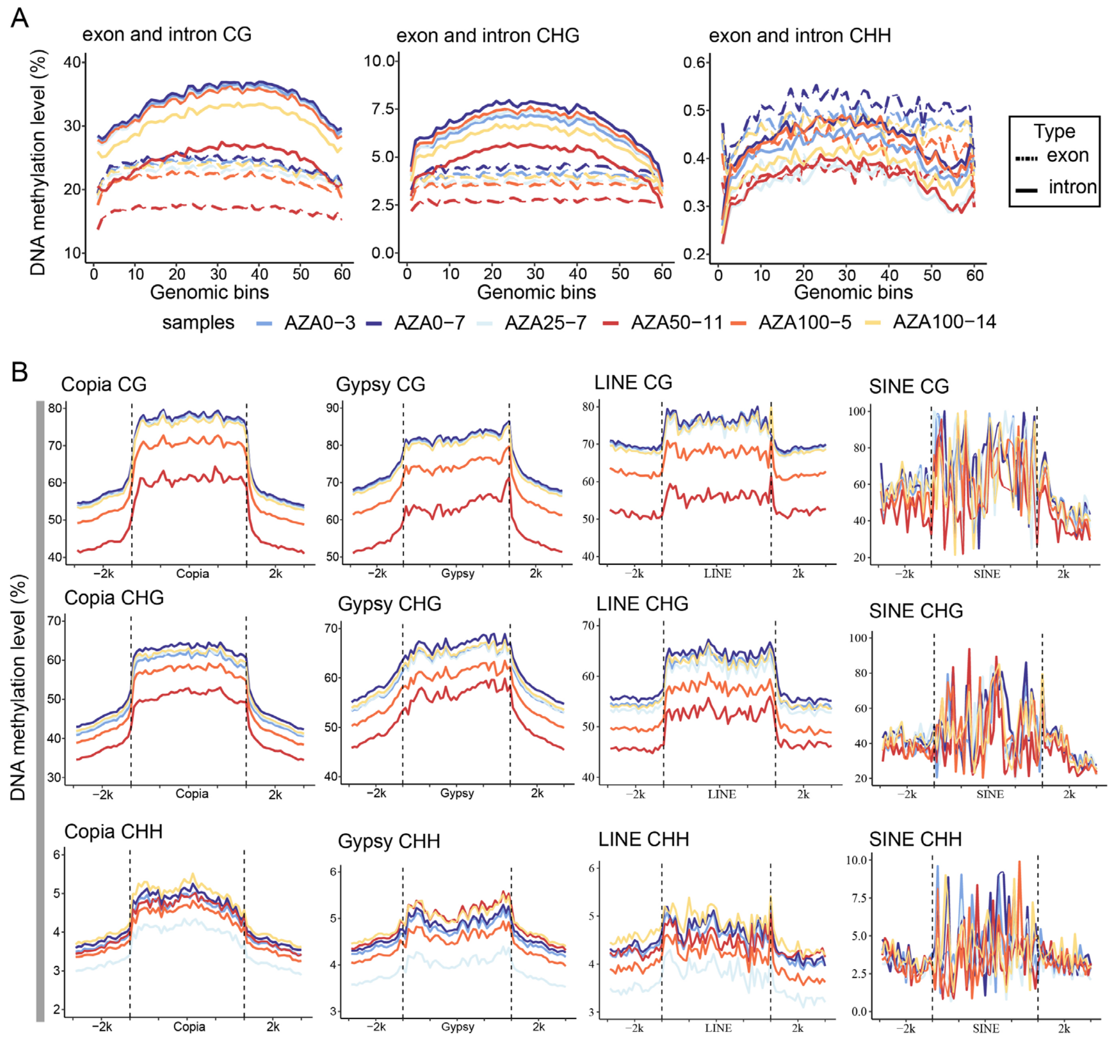

Supplementary Figure S8. DNA methylation levels in exon and intron of protein coding genes and four types of transposable elements (TEs). (A), Gene, **(B),** TEs. The data was present in CG, CHG, and CHH contexts. Each intron and exon were equally divided into 60 bins.

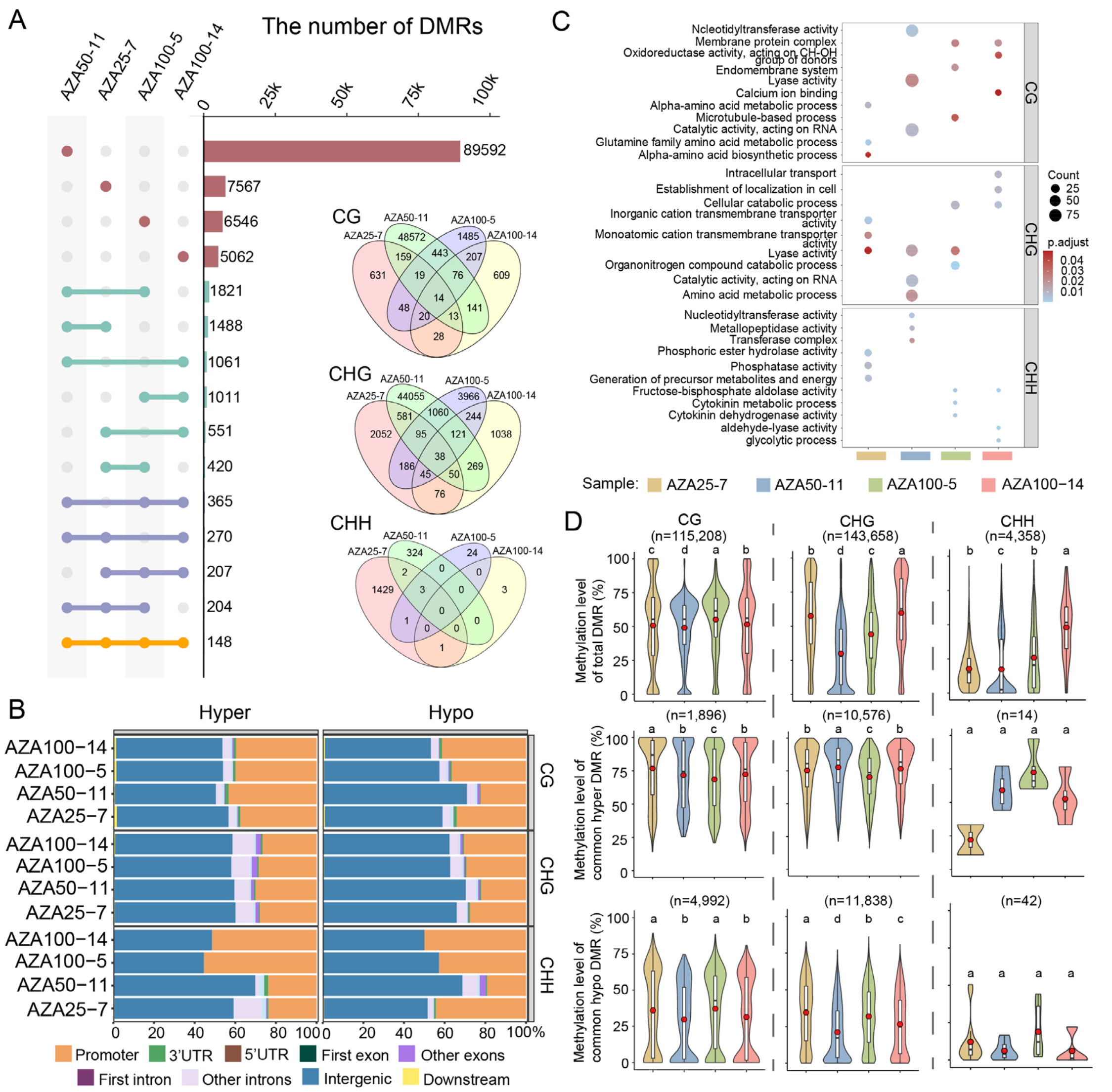

Supplementary Figure S9. Comparative methylome analysis of epimutants revealed epigenetic divergence. Sample AZA0-3 was used as control in this data set. (**A**), Intersection patterns of DMRs across four epimutants. Horizontal bars with colored nodes (Upset plot) denote shared DMR subsets, with the inset quantifying shared hypomethylated DMRs (hypo-DMRs) in CG, CHG, and CHH sequence contexts. (**B**), Proportions of hyper (left) and hypo (right) DMRs across regions of diverse features. (**C**), Functional enrichment analysis of genes overlapping with hypo-DMRs. (**D**), Distributions of methylation levels of DMR in epimutants in three contexts. Upper panel, total DMR; Middle panel, hyper DMR; Bottom panel, hypo DMR. Distinct letters indicate statistically divergent groups (Mann-Whitney U test, two-sided, $p < 0.05$). In the boxplots, the center lines represent the medians, the box limits correspond to the 25th and 75th percentiles, the whiskers extend to 1.5 times the interquartile ranges, and individual points indicate outliers.

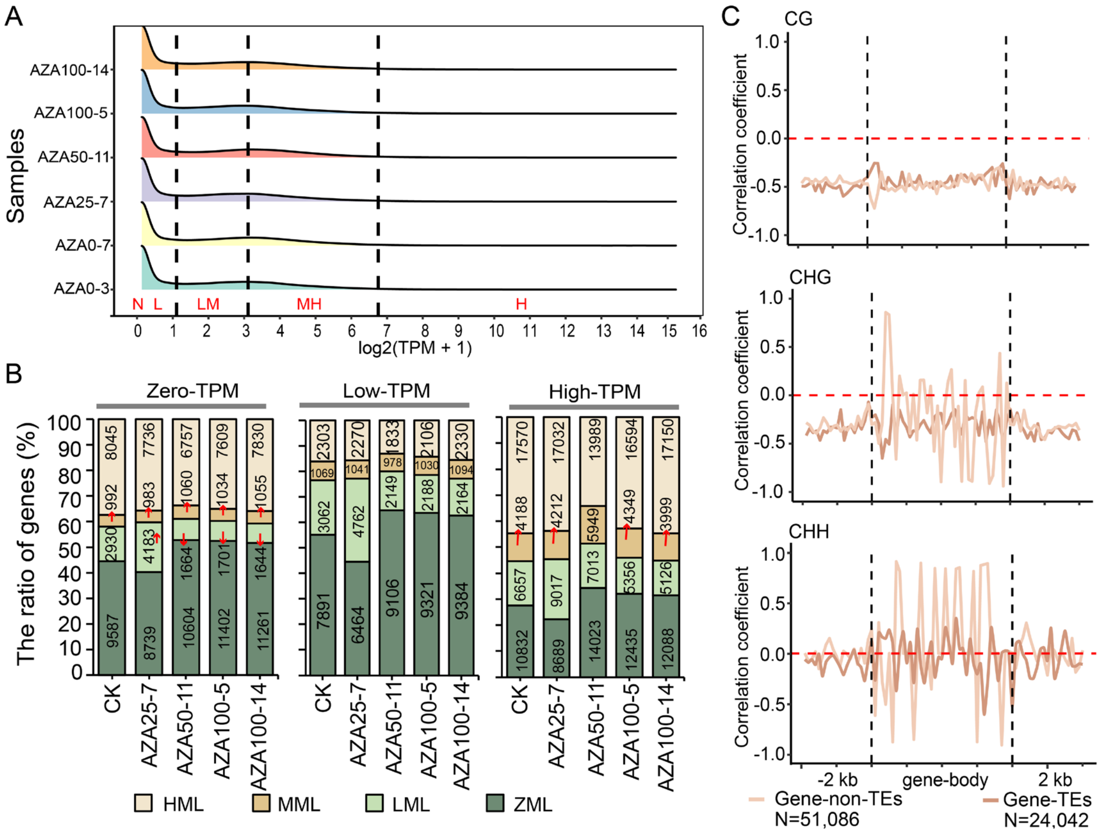

Supplementary Figure S10. Correlations of gene expression levels and DNA methylation levels. (**A**), Categorization of genes based on expression levels. N: TPM = 0; L: 0 < TPM ≤ 1; LM: 1 < TPM ≤ 7; MH: 7 < TPM ≤ 100; H: 100 < TPM.

(**B**), Portions of genes with ranked DNA methylation levels for genes with zero, low and high TPMs. Categories of methylation levels: ZML (zero methylation level, methylation level=0), LML (low methylation level, (0,10]), MML (middle methylation level, (10,20]), and HML (high methylation level, (20,100]). Categories of expression levels: Zero-TPM (N group), Low-TPM (L group), High-TPM (LM, MH and H groups). The red arrows represent the values for the column blocks with small sizes that are hard for direct labelling. (**C**), Averaged PCCs between DNA methylation levels of genomic bins and gene expression levels for Gene-non-TEs and Gene-TEs. The values were plotted alongside relative genomic positions to each gene.

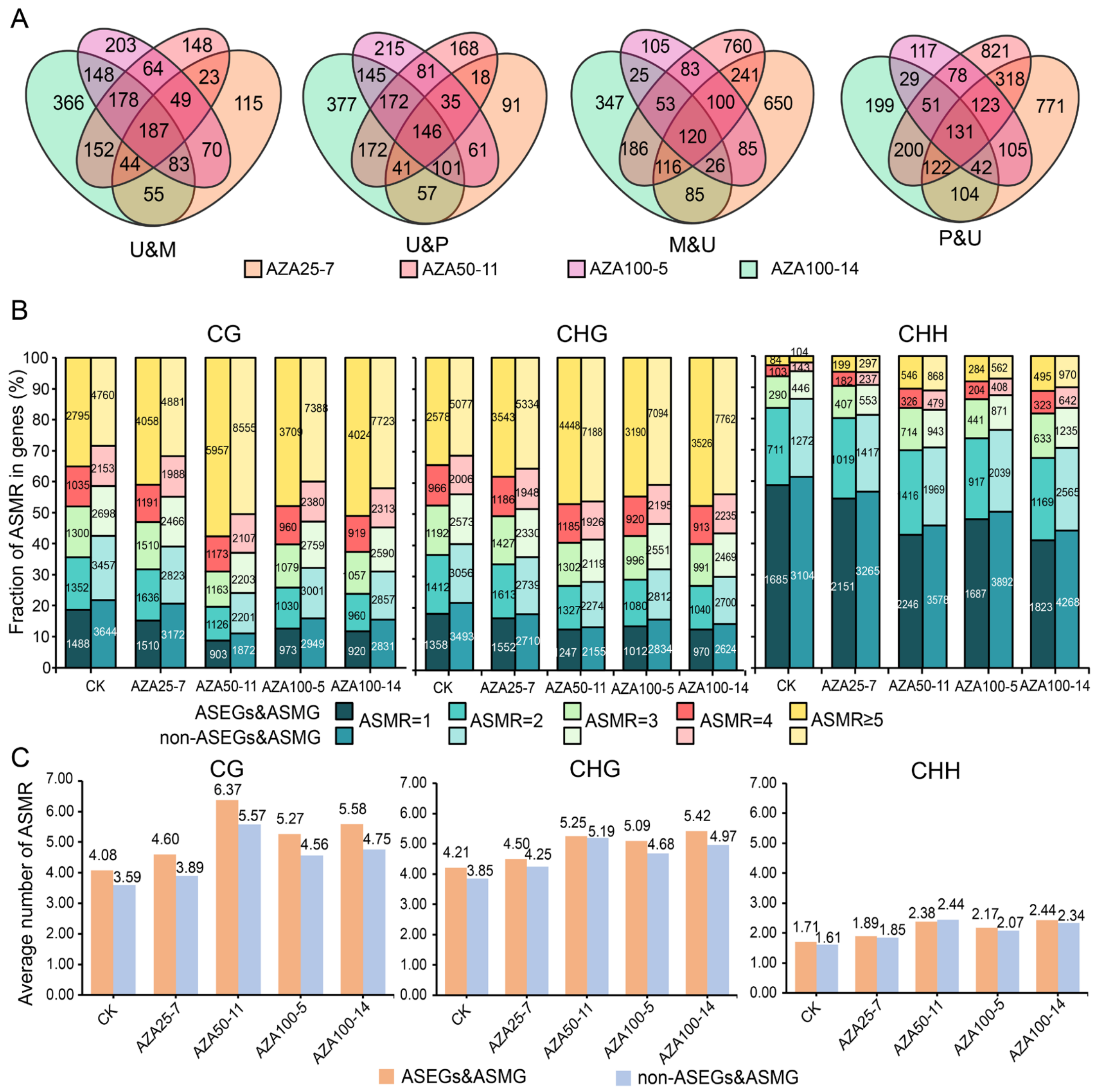

Supplementary Figure S11. Allele-specific biased gene expression and DNA methylation in epimutants. (**A**), The Venn diagrams showing overlapped ASEGs of four categories of epimutants (U&M, U&P, M&U, P&U, these categories same as in Figure 6B, the first and second letters indicated the directions of ASEGs in epimutant and control, respectively). (**B**), Disruptions of allele pairs with diverse ASMRs for ASEGs and non-ASEGs. The allele pairs were categorized into five classes (ASMR=1, ASMR=2, ASMR=3, ASMR=4 and ASMR ≥5) based on numbers of overlapped ASMRs. The ASMRs were detected in three contexts for control plants and epimutants. (**C**), Average number of ASMR in ASEGs and non-ASEGs in three contexts.

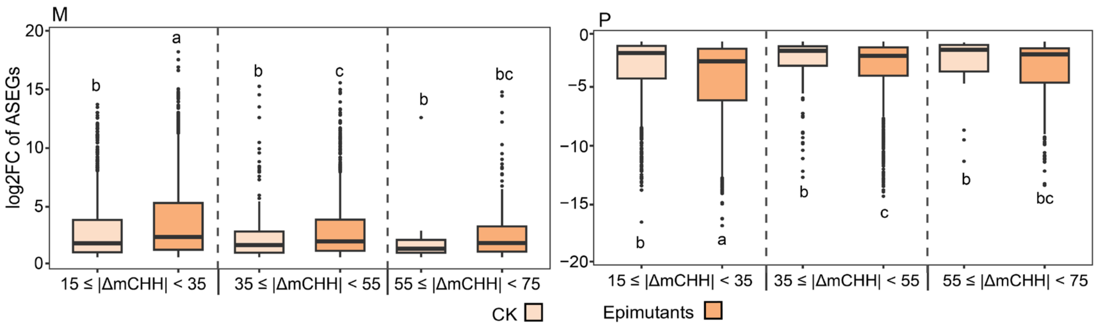

Supplementary Figure S12. Fold changes between alleles of gene locus with allele-specific methylation at three grades of divergence in CHH contexts. Letters denote statistically distinct groups (Wilcoxon.test, $p < 0.01$). In the boxplots, the center lines represent the medians, the box limits correspond to the 25th and 75th percentiles, the whiskers extend to 1.5 times the interquartile ranges, and individual points indicate outliers.

Supplementary Tables

Supplementary Table S1. Statistics of differentiation time and differentiation rate of leaf explants from NL895 on medium supplemented with 5-Aza.

Treatment (5-Aza concentration, μM)	Differentiation time (d)	Leaf discs cultured	Differentiation rate (%)	Numbers of regenerated plants
0	14±2	34	91.18	37
5	12±1	35	97.14	44
10	12±3	36	94.44	32
25	17±2	34	94.12	40
50	19±1	34	85.29	31
100	22±3	35	88.57	26
200	26±2	36	54.55	0
1000	40±1	35	5.71	0

Supplementary Table S2. Categories of epimutants with diverse phenotypes.

Mutant phenotype	Traits	Mutant strains	Number of plants
Leaf morphology	Increased crenate leaf margins	AZA5-9, AZA100-5, AZA100-11	3
Leaf morphology	Round leaves	AZA5-9, AZA50-11	2
Leaf morphology	Wrinkled leaves	AZA50-11, AZA100-3	2
Leaf morphology	Leathery leaves	AZA100-12, AZA100-14	2
Leaf color	White leaves	AZA25-11	1
Leaf color	Yellow leaves	AZA10-9, AZA10-23	1
Others	Cuneate basal leaves	AZA100-5	1
Others	Clustered leaves	AZA25-7, AZA50-19	2
Others	Short internodes	AZA50-11	1
Total	-	-	15

Supplementary Table S3. Sites and lengths of the telomeres on hap1.

Chromosome	Left Start (bp)	Left End (bp)	Left Length (bp)	Right Start (bp)	Right End (bp)	Right Length (bp)
1	1	11,521	11,521	48,502,167	48,533,723	31,557
2	1	13,792	13,792	-	-	-
3	1	20,623	20,623	24,604,132	24,616,090	11,959
4	1	12,364	12,364	22,569,260	22,584,680	15,421
5	1	14,293	14,293	24,132,888	24,146,925	14,038
6	1	10,178	10,178	-	-	-
7	1	17,948	17,948	15,989,431	16,003,574	14,144
8	1	18,139	18,139	-	-	-
9	1	29,243	29,243	14,047,537	14,055,596	8,060
10	1	35,781	35,781	22,037,478	22,038,363	886
11	-	-	-	21,890,310	21,906,833	16,524
12	1	8,603	8,603	13,731,719	13,749,086	17,368
13	1	18,080	18,080	16,765,581	16,775,139	9,559
14	1	18,141	18,141	-	-	-
15	1	12,168	12,168	14,790,482	14,804,744	14,263
16	1	14,659	14,659	14,875,677	14,886,877	11,201
17	1	12,923	12,923	15,356,437	15,368,052	11,616
18	1	8,055	8,055	16,617,258	16,626,016	8,759
19	1	204	204	-	-	-

Supplementary Table S4. Sites and lengths of the telomeres on hap2.

Chromosome	Left Start (bp)	Left End (bp)	Left Length (bp)	Right Start (bp)	Right End (bp)	Right Length (bp)
1	1	4,764	4,764	50,019,534	50,038,316	18,783
2	1	24,059	24,059	24,842,085	24,845,649	3,565
3	1	9,164	9,164	21,314,763	21,341,940	27,178
4	1	31,478	31,478	-	-	-
5	1	10,874	10,874	23,179,467	23,189,249	9,783
6	1	9,262	9,262	26,759,680	26,774,003	14,324
7	1	20,479	20,479	15,727,586	15,744,483	16,898
8	-	-	-	-	-	-
9	1	25,068	25,068	13,316,980	13,337,690	20,711
10	1	15,185	15,185	23,059,900	23,071,619	11,720
11	1	22,760	22,760	18,485,068	18,513,497	28,430
12	1	14,428	14,428	14,143,303	14,162,166	18,864
13	1	19,287	19,287	-	-	-
14	1	22,168	22,168	-	-	-
15	1	23,865	23,865	14,865,935	14,869,490	3,556
16	1	21,133	21,133	14,642,700	14,678,315	35,616
17	1	32,693	32,693	15,996,551	16,005,337	8,787
18	1	1,536	1,536	-	-	-
19	-	-	-	16,573,037	16,578,409	5,373

Supplementary Table S5. Repetitive elements identified in the hap1 and hap2 assemblies.

Type	Number	NL895_hap1 Length (bp)	Percentage (%)	Number	NL895_hap2 Length (bp)	Percentage (%)
SINEs	2,281	405,799	0.1	6,147	971,130	0.24
LINEs	2,997	1,635,647	0.41	3,144	2,628,162	0.66
LTR elements	71,857	56,883,681	14.24	87,442	61,923,619	15.46
DNA elements	34,193	21,329,910	5.34	36,223	18,018,904	4.5
Unclassified interspersed repeats	112,593	59,198,738	14.82	103,232	56,231,944	14.04
Simple repeats	143,763	6,188,001	1.55	138,750	6,158,589	1.54
Small RNA	2,736	2,464,988	0.62	7,614	2,840,575	0.71
Satellites	1,249	3,430,698	0.86	746	488,086	0.12
Low complexity	31,387	1,558,007	0.39	31,839	1,578,884	0.39
Total	403,056	153,095,469	38.33	415,137	150,839,893	37.66

Supplementary Table S6. Statistics of protein-coding gene with annotated functions using diverse databases.

Method	Database	hap1		hap2	
		Number	Percent (%)	Number	Percent (%)
DIAMOND	*P.trichocarpa*	32,152	84.43	31,994	86.36
	P.trichocarpa	28,720	75.42	28,824	77.80
	O.sativa	27,465	72.12	27,597	74.49
	Swiss Prot	24,607	64.62	24,669	66.59
	NR	35,041	92.02	34,839	94.04
eggNOG-mapper	eggNOG	31,174	81.86	31,179	84.16
	GO	18,439	48.42	18,497	49.93
	KEGG_KO	12,673	33.28	12,694	34.26
	KEGG_Pathway	11,079	29.09	11,165	30.14
Pfam		25,341	66.54	25,614	69.14

Supplementary Table S7. BUSCO analysis for annotated transcriptomes of two haplotypes and publishes monoploid assemblies.

Type	Haplotype1		Haplotype2		NL895_Monoploid	
	Number	Percentage (%)	Number	Percentage (%)	Number	Percentage (%)
Complete BUSCOs (C)	1,322	96.1	1,332	96.9	1,301	94.6
Complete and single-copy BUSCOs (S)	1,099	79.9	1,118	81.3	1,080	78.5
Complete and duplicated BUSCOs (D)	223	16.2	214	15.6	221	16.1
Fragmented BUSCOs (F)	30	2.2	16	1.2	28	2
Missing BUSCOs (M)	23	1.7	27	1.9	46	3.4
Total BUSCO groups searched	1,375		1,375		1,375	

Supplementary Table S8. Summary of identified genomic variations.

Variation type	hap2 vs hap1			Monoploid vs hap1			I-69 vs hap1		
	Count	Length (hap1)	Length (hap2)	Count	Length (hap1)	Length (Monoploid)	Count	Length (hap1)	Length (*P. deltoides I-69*)
**Structural Variations**									
Syntenic regions	8,424	281,466,403	282,203,634	2,501	343,973,529	344,149,731	754	353,394,321	358,734,783
Inversions	208	8,426,932	8,784,840	82	2,945,771	2,380,564	64	10,065,742	10,037,525
Translocations	7,486	13,391,181	13,432,004	1,997	5,450,979	5,317,766	487	15,115,185	14,559,539
Duplications (reference)	980	1,576,829	-	211	371,694	-	64	3,874,900	-
Duplications (query)	1,122	-	1,730,067	260	-	517,183	111	-	1,158,125
Not aligned (reference)	14,893	94,588,102	-	4,218	46,420,411	-	763	22,995,855	-
Not aligned (query)	14,987	-	94,044,469	4,265	-	40,860,618	1,114	-	30,865,177
Copygains	1,923	-	2,544,358	969	-	1,758,085	1,117	-	11,281,111
Copylosses	1,719	2,463,907	-	871	2,400,236	-	547	2,319,785	-
Highly diverged	39,277	80,038,860	81,651,454	19,085	42,497,626	42,694,428	4,530	7,468,782	5,036,673
Tandem repeats	204	111,602	110,378	106	225,033	207,253	165	474,783	362,757
**Short Variations**									
SNPs	7,727,425	7,727,425	7,727,425	3,373,714	3,373,714	3,373,714	1,913,864	1,913,864	1,913,864
Insertions	736,567	-	4,887,196	323,281	-	2,540,887	270,862	-	2,550,817
Deletions	715,628	5,423,821	-	324,154	2,731,581	-	328,308	2,281,616	-

Note: In the analysis, the sequences of hap1 assembly was used as reference.

Variation type	Structural Variations					
	Monoploid vs hap2			I-69 vs hap2		
	Count	Length (hap2)	Length (Monoploid)	Count	Length (hap2)	Length (*P. deltoides I-69*)
Syntenic regions	3,405	330,269,732	328,976,438	8,264	273,514,010	273,666,264
Inversions	122	9,668,705	8,212,414	231	16,883,544	18,265,268
Translocations	2,803	7,270,120	7,248,806	7,919	19,912,847	20,173,200
Duplications (reference)	326	1,561,083	-	1,274	3,028,034	-
Duplications (query)	368	-	681,727	1,668	-	3,267,985
Not aligned (reference)	5,876	51,872,311	-	15,117	90,248,789	-
Not aligned (query)	5,935	-	48,103,724	16,150	-	101,782,838
Copygains	942	-	1,924,805	1,675	-	2,638,648
Copylosses	1,047	2,768,038	-	1,871	2,639,814	-
Highly diverged	21,349	50,859,914	48,374,522	39,109	87,416,992	88,624,443
Tandem repeats	113	169,486	144,638	189	97,845	104,802
**Short Variations**						
SNPs	3,992,434	3,992,434	3,992,434	7,755,138	7,755,138	7,755,138
Insertions	387,380	-	3,276,078	825,138	-	5,470,949
Deletions	356,624	2,819,086	-	705,967	4,912,250	-

Note: In the analysis, the sequences of hap2 assembly was used as reference.

Variation type	Structural Variations					
	Monoploid vs I-69			*P.nigra* vs I-69		
	Count	Length (*P. deltoides I-69*)	Length (Monoploid)	Count	Length (*P. deltoides I-69*)	Length (*P.nigra*)
Syntenic regions	3,226	325,281,412	323,625,554	8,163	276,326,563	275,022,045
Inversions	140	14,341,691	15,866,425	222	13,057,926	12,640,768

Variation type	Structural Variations					
	Monoploid vs I-69			*P.nigra* vs I-69		
	**Count**	**Length (*P. deltoides I-69*)**	**Length (Monoploid)**	**Count**	**Length (*P. deltoides I-69*)**	**Length (*P.nigra*)**
Translocations	2,783	16,020,467	16,089,098	7,930	20,714,111	20,585,458
Duplications (reference)	395	-	707,199	1,639	4,047,132	-
Duplications (query)	623	2,008,821	-	1,275	-	2,206,509
Not aligned (reference)	5,298	-	40,875,454	15,934	103,856,528	-
Not aligned (query)	5,964	59,726,721	-	15,124	-	106,127,164
Copygains	1,422	6,229,501	-	1,829	-	2,382,669
Copylosses	1,184	-	2,517,704	1,695	2,706,822	-
Highly diverged	21,160	47,278,097	50,749,419	39,237	87,603,481	86,307,368
Tandem repeats	177	207,041	228,165	199	105,246	102,051
**Short Variations**						
SNPs	4,422,657	4,422,657	4,422,657	7,722,859	7,722,859	7,722,859
Insertions	508,238	4,075,540	-	781,447	-	5,034,003
Deletions	494,408	-	3,391,839	722,538	5,284,124	-

Note: In the analysis, the sequences of *P. deltoides I-69* assembly was used as reference.

Supplementary Table S9. Summary of total and mapped WGBS reads.

Sample	Total reads	Clean reads	Clean ratio (%)	Unique mapping reads	Read length(bp)	Data size(bp)	Sequencing depth	Bisulfite conversion rate (%)	Unique mapping rate (%)
AZA0-3-1	74,034,015	72,698,158	98.20	54,194,931	150	21,343,849,848	26.68	95.43%	74.50%
AZA0-3-2	67,719,203	67,263,656	99.33	45,581,303	150	19,896,228,635	24.87	95.53%	67.80%
AZA0-7-1	80,430,830	79,166,157	98.43	56,489,890	150	23,256,333,838	29.07	95.32%	71.40%
AZA0-7-2	67,036,255	66,621,871	99.38	44,178,659	150	19,710,499,288	24.64	95.54%	66.30%
AZA100-14-1	72,304,013	71,054,397	98.27	50,909,471	150	20,869,207,268	26.09	95.54%	71.60%
AZA100-14-2	67,331,505	67,010,424	99.52	47,365,911	150	19,873,875,424	24.84	95.33%	70.70%
AZA100-5-1	73,280,343	72,031,419	98.30	50,174,383	150	21,153,184,511	26.44	95.82%	69.70%
AZA100-5-2	66,849,483	66,500,683	99.48	43,500,919	150	19,712,558,141	24.64	95.68%	65.40%
AZA25-7-1	67,661,421	66,542,472	98.35	46,210,844	150	19,547,893,603	24.44	95.75%	69.40%
AZA25-7-2	67,813,481	67,390,024	99.38	43,317,424	150	19,925,237,949	24.91	95.61%	64.30%
AZA50-11-1	70,803,427	69,498,500	98.16	49,709,487	150	20,382,654,201	25.48	96.22%	71.50%
AZA50-11-2	67,007,611	66,551,278	99.32	46,771,993	150	19,693,244,503	24.62	96.08%	70.30%

Supplementary Table S10. Hyper and hypo DMRs in comparison to AZA0-7 in three contexts.

Methylation contexts	Methylation patterns	AZA0-3	AZA25-7	AZA50-11	AZA100-5	AZA100-14
Distinct CG	hyper	-	1,218	1,635	422	555
	hypo	-	1,563	58,744	3,229	661
Distinct CHG	hyper	-	2,337	3,611	1,072	2,642
	hypo	-	4,052	53,934	11,801	2,958
Distinct CHH	hyper	-	101	2,860	188	1,115
	hypo	-	1,670	14,631	738	209
Shared CG with CK	hyper	-	1,087	859	188	258
	hypo	-	1,245	1,892	363	217
Shared CHG with CK	hyper	-	1,107	934	453	826
	hypo	-	2,332	5,239	2,788	1,287
Shared CHH with CK	hyper	-	12	51	11	40
	hypo	-	76	128	60	14
all CG	hyper	3,859	2,305	2,494	610	813
	hypo	4,690	2,808	60,636	3,592	878
all CHG	hyper	4,939	3,444	4,545	1,525	3,468
	hypo	16,164	6,384	59,173	14,589	4,245
all CHH	hyper	412	113	2,911	199	1,155
	hypo	690	1,746	14,759	798	223
all DMR	hyper	9,210	5,862	9,950	2,334	5,436
	hypo	21,544	10,938	134,568	18,979	5,346
	hyper + hypo	30,754	16,800	144,518	21,313	10,782
all DMR (%)	hyper	29.95%	34.89%	6.88%	10.95%	50.42%
	hypo	70.05%	65.11%	93.12%	89.05%	49.58%
all CG (%)	hypo	21.77%	25.67%	45.06%	18.93%	16.42%
all CHG (%)	hypo	75.03%	58.37%	43.97%	76.87%	79.41%
all CHH (%)	hypo	3.20%	15.96%	10.97%	4.20%	4.17%

Supplementary Table S11. Hyper and hypo DMRs in comparison to AZA0-3 in three contexts.

Methylation contexts	Methylation patterns	AZA0-7	AZA25-7	AZA50-11	AZA100-5	AZA100-14
Distinct CG	hyper	-	950	1,007	684	1,174
	hypo	-	932	49,437	2,312	1,108
Distinct CHG	hyper	-	4,305	3,901	2,043	4,552
	hypo	-	3,123	46,269	5,755	1,881
Distinct CHH	hyper	-	181	159	9	33
	hypo	-	1,436	329	28	4
Shared CG with CK	hyper	-	477	377	991	1,575
	hypo	-	362	1,038	1,343	1,488
Shared CHG with CK	hyper	-	3,478	2,515	2,805	5,220
	hypo	-	654	1,367	1,367	1,260
Shared CHH with CK	hyper	-	33	3	5	9
	hypo	-	65	3	10	3
all CG	hyper	4,690	1,427	1,384	1,675	2,749
	hypo	3,859	1,294	50,475	3,655	2,596
all CHG	hyper	16,164	7,783	6,416	4,848	9,772
	hypo	4,939	3,777	47,636	7,122	3,141
all CHH	hyper	690	214	162	14	42
	hypo	412	1,501	332	38	7
all DMR	hyper	21,544	9,424	7,962	6,537	12,563
	hypo	9,210	6,572	98,443	10,815	5,744
	hyper + hypo	30,754	15,996	106,405	17,352	18,307
all DMR (%)	hyper	70.05%	58.91%	7.48%	37.67%	68.62%
	hypo	29.95%	41.09%	92.52%	62.33%	31.38%
all CG (%)	hypo	41.90%	19.69%	51.27%	33.80%	45.19%
all CHG (%)	hypo	53.63%	57.47%	48.39%	65.85%	54.68%
all CHH (%)	hypo	4.47%	22.84%	0.34%	0.35%	0.12%

Supplementary Table S12. The enrichment analysis of DMGs in total DEGs between epimutants and control plants.

The analysis was performed using the one-tailed Fisher's exact test in three contexts.

Contexts	Samples	Group	DMGs	non-DMGs	*p* value
CG	AZA25-7	DEGs	42	804	0.001
		non-DEGs	2,096	72,184	
	AZA50-11	DEGs	1,339	3,615	0.034
		non-DEGs	18,129	52,043	
	AZA100-5	DEGs	1	66	0.928
		non-DEGs	2,893	72,166	
	AZA100-14	DEGs	81	2,733	0.084
		non-DEGs	1,768	70,544	
CHG	AZA25-7	DEGs	56	790	0.581
		non-DEGs	5,016	69,264	
	AZA50-11	DEGs	1,152	3,802	0.012
		non-DEGs	15,345	54,827	
	AZA100-5	DEGs	3	64	0.967
		non-DEGs	7,424	67,635	
	AZA100-14	DEGs	198	2,616	0.033
		non-DEGs	4,452	67,860	
CHH	AZA25-7	DEGs	35	811	0.001
		non-DEGs	1,657	72,623	
	AZA50-11	DEGs	309	4,645	0.000
		non-DEGs	2,902	67,270	
	AZA100-5	DEGs	-	67	1.000
		non-DEGs	501	74,558	
	AZA100-14	DEGs	44	2,770	0.002
		non-DEGs	698	71,614	
